# Isolation and Characterization of Neural Crest-Derived Stem Cells from Dental Pulp of Neonatal Mice

**DOI:** 10.1371/journal.pone.0027526

**Published:** 2011-11-08

**Authors:** Kajohnkiart Janebodin, Orapin V. Horst, Nicholas Ieronimakis, Gayathri Balasundaram, Kanit Reesukumal, Busadee Pratumvinit, Morayma Reyes

**Affiliations:** 1 Department of Oral Health Sciences, School of Dentistry, University of Washington, Seattle, Washington, United States of America; 2 Departments of Dental Public Health Sciences and Endodontics, School of Dentistry, University of Washington, Seattle, Washington, United States of America; 3 Department of Pathology, Institute for Stem Cell and Regenerative Medicine, School of Medicine, University of Washington, Seattle, Washington, United States of America; 4 Department of Anatomy, Faculty of Dentistry, Mahidol University, Bangkok, Thailand; 5 Department of Clinical Pathology, Faculty of Medicine Siriraj Hospital, Mahidol University, Bangkok, Thailand; University of Southern California, United States of America

## Abstract

Dental pulp stem cells (DPSCs) are shown to reside within the tooth and play an important role in dentin regeneration. DPSCs were first isolated and characterized from human teeth and most studies have focused on using this adult stem cell for clinical applications. However, mouse DPSCs have not been well characterized and their origin(s) have not yet been elucidated. Herein we examined if murine DPSCs are neural crest derived and determined their in vitro and in vivo capacity. DPSCs from neonatal murine tooth pulp expressed embryonic stem cell and neural crest related genes, but lacked expression of mesodermal genes. Cells isolated from the *Wnt1-Cre/R26R-LacZ* model, a reporter of neural crest-derived tissues, indicated that DPSCs were *Wnt1*-marked and therefore of neural crest origin. Clonal DPSCs showed multi-differentiation in neural crest lineage for odontoblasts, chondrocytes, adipocytes, neurons, and smooth muscles. Following in vivo subcutaneous transplantation with hydroxyapatite/tricalcium phosphate, based on tissue/cell morphology and specific antibody staining, the clones differentiated into odontoblast-like cells and produced dentin-like structure. Conversely, bone marrow stromal cells (BMSCs) gave rise to osteoblast-like cells and generated bone-like structure. Interestingly, the capillary distribution in the DPSC transplants showed close proximity to odontoblasts whereas in the BMSC transplants bone condensations were distant to capillaries resembling dentinogenesis in the former vs. osteogenesis in the latter. Thus we demonstrate the existence of neural crest-derived DPSCs with differentiation capacity into cranial mesenchymal tissues and other neural crest-derived tissues. In turn, DPSCs hold promise as a source for regenerating cranial mesenchyme and other neural crest derived tissues.

## Introduction

Dental pulp is a loose connective tissue which contains heterogenous cell populations located in the central part of the tooth [1]. Dental pulp stem cells (DPSCs), first discovered by Gronthos and Shi, were isolated from human adult teeth and characterized as highly proliferative cells with self renewal, multi-differentiation *in vitro*, and capacity to form dentin/pulp-like structures *in vivo* [2], [3]. Initial studies described heterogeneity of DPSCs. Subsequent studies attempted to use clonogenic assays and stem cell markers to purify DPSC populations [3], [4]. Nevertheless, the multi-differentiation of DPSCs *in vitro* and *in vivo* has been variable as described in several reports [3], [5], [6].

Differential Notch expression was observed in various locations within the dental pulp. Notch signaling is important for stem cell determination, which implies that dental pulp may harbor several stem cell subpopulations with different capacity and origins [7]. Alternatively all DPSCs share a common developmental origin but their niche and location dictates their behavior. The expression and function of the Eph/ephrin molecules on DPSCs, which play an essential role in the neural crest migration, suggests that neural crest contributes to DPSCs [8]. The *Wnt1-Cre/R26R-LacZ* used to trace neural crest developmental origin has indicated that the majority of dental pulp cells are *Wnt1*-marked, with some contribution from unmarked *Wnt1*- non-neural crest cells [9]. Recently, stem cell populations in adult human dental pulp have been described based on low-affinity nerve growth factor receptor (LNGFR) and β1-integrin expressions, suggesting that the former population is neural crest-derived [10]. In a separate study stem cells isolated from rat embryonic mandibular processes, of neural crest origin, showed multi-differentiation to neural and mesenchymal lineages suggesting DPSCs retain the differentiation capacity of embryonic progenitors [11]. Collectively, these reports urged us to investigate the origin of DPSCs.

Cranial neural crest (CNC) cells migrate to the first branchial arch and participate in differentiation of dental mesenchyme to develop dental pulp and giving rise to dentin-forming cells, odontoblasts [12]. In addition, CNCs show multipotent capacity by giving rise to skeletal and connective tissues of head and neck, as well as, neuron and glial cells in cranial ganglia [13]. During tooth development dental pulp tissue is derived from neural crest and non-neural crest tissues [9]. Although neural crest contributes to major cell constituents in dental pulp, the origin of DPSCs is still unclear. Herein, we describe neural crest-derived DPSCs from *Tie2-GFP* and *Wnt1-Cre/R26R-LacZ* neonatal mice and demonstrate their highly proliferative capacity and multi-differentiation in neural crest-lineage *in vitro* and *in vivo*. Furthermore, the comparison of the niches and mineralized matrix generated by bone marrow stromal cells (BMSCs) and DPSCs when transplanted *in vivo* illustrates main differences in dentinogenesis and osteogenesis and the role of pericytes and microvessels during these processes.

## Results

### DPSC isolation and culture

Dental pulp was isolated from neonatal murine mandibular molar teeth because these developing teeth have not formed roots yet (Figures S1A, S1B), which makes pulp dissection feasible. Since the dental pulp is a highly vascularized tissue, the *Tie2-GFP* mouse model was used to determine the contribution of endothelial and hematopoietic cells in culture by screening for GFP expression driven by the *Tie2* promoter [14]. Following constant monitoring for the presence of GFP positive cells we concluded all dental pulp cultures derived from *Tie2-GFP* were completely negative for GFP, indicating our cultures did not contain endothelial and/or hematopoietic cells (data not shown). In addition, cells harvested from early cultures were negative for endothelial expressed genes *vWF (von Willebrand Factor)*, *CD31 (PECAM)*, angiopoeitin receptors *Tie1* and *Tie2* and *Ve-Cadherin* by RT-PCR (data not shown).

To identify highly proliferative populations, we cultured freshly isolated dental pulp mononuclear cells at low density, 1000 cells/cm^2^, in stem cell media with 2% serum as a selective condition to enrich for stem cell outgrowth, and 5% CO_2_/O_2_ to more accurately replicate physiological conditions [15]. Within two days in culture, cells began to proliferate and form colonies (Figure S1C). By day 10 in culture, cells formed large and confluent colonies (Figure S1D), that were split and subsequently passaged approximately every 3–4 days. In each passage, we used a 1∶4 dilution as the standard ratio for cell expansion. Cells were cultured until passage 14 (day 90), and grew at a consistent and steady proliferation rate without signs of senescence, suggesting that DPSCs are highly proliferative. DPSCs from three independent isolations were characterized; each of which demonstrated similar growth pattern and proliferation rate. The morphology of cultured cells was heterogeneous in early culture but most appeared spindle-shaped (Figure S1E).

### DPSCs express stem cell and neural crest–related genes

**Figure 1:**
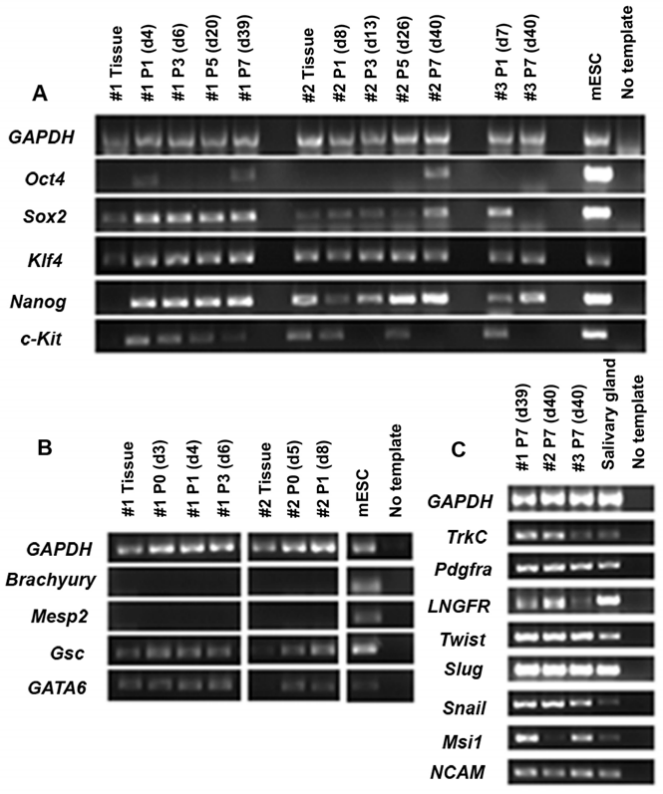
Gene profile of DPSC cultures. Gene expression of fresh dental pulp and DPSCs cultured several passages from three isolations (#1, #2 and #3) are shown. (A) Stem cell genes, *Oct4*, *Sox2*, *Klf4*, *Nanog* and *c-Kit,* were observed; however, *Oct4* was inconsistent and transient from early to late passages. (B) In contrast to neural crest developmental genes *Gsc* and G*ATA6,* early mesodermal developmental genes *Brachyury* and *Mesp2* were not expressed. (C) Neural crest-related genes *TrkC, Pdgfra, LNGFR, Twist, Slug, Snail, Msi1*, and *NCAM*, were continuously expressed in all three non-clonal DPSCs. RNA isolated from mouse embryonic stem cells (mESCs) and salivary gland were used as positive control for the expression of stem cell/early developmental genes and neural crest genes, respectively. The PCR reaction without template was used as negative control.

To gain insights into the stem cell properties and possible origin of DPSCs, we surveyed the expression of stem cell genes as well as neural crest and mesodermal genes in freshly isolated dental pulp tissue and three DPSC lines generated from early to late cultures. RT-PCR (Figure 1A) showed absence of *Oct4* in both fresh tissue and cells in early culture, while *Sox2* was generally expressed. However, *Oct4* was expressed in the late culture (passage 7) by two DPSC lines. Surprisingly, *Klf4* was highly expressed and maintained even in late passages by all three DPSC lines. *Klf4* and *Sox2* are two of four pluripotency genes required to generate inducible pluripotent stem cells [16]. Moreover, *Nanog* which is important for the maintenance of pluripotency in embryonic stem cells was up-regulated in culture [17]. In turn, expression of these pluripotency genes suggests the presence of a primitive stem cell population in our cultures. DPSCs also expressed variable levels of *c-Kit* which has been reported to play important roles for cell survival, proliferation and differentiation of multiple types of stem cells including neural crest stem cells destined to form melanocytes [18].

To characterize the potential origin of DPSCs, we examined by RT-PCR the expression of early developmental genes in DPSCs (Figure 1B). As expected, we observed a complete absence of genes expressed by mesodermal cells during development, *Brachyury* and *Mesp2* [19], [20]. DPSCs did however express genes associated with neural crest development, *Goosecoids (Gsc)* and *GATA6* [21], [22]. In addition, DPSCs expressed embryonic neural crest and neuronal stem/precursor cell associated genes (Figures 1C, S2), *tyrosine kinase C* (*TrkC*), *platelet-derived growth factor receptor-alpha* (*Pdgfra*), *LNGFR*, *Twist*, *Snail*, *Slug, Sox10, neural cell adhesion molecule* (*NCAM),* and *Musashi1* (*Msi1*) [23]–[27]. We further examined the presence of neural crest associated proteins in DPSCs by immunocytochemistry. Undifferentiated cells showed positive staining for the pluripotent transcription factor, KLF4 and neural crest-related factors, MSI1 and SOX10 (Figure S2). Although we cannot rule out the existence of non-neural crest cells in these non-clonal cultures, these results suggest that a neural crest derived population existed in our DPSC cultures.

### DPSCs in vitro differentiate into neural crest-lineage cells

During development of head and neck, neural crest cells migrate to the craniofacial region and give rise to neurons, glia, bone, cartilage, and connective tissues, including odontoblasts [9], [13]. Therefore, *in vitro* differentiation of DPSCs into neural-crest lineages supports the hypothesis that DPSCs maintain embryonic neural crest potential. We studied the capacity of DPSCs to differentiate into neural crest-lineage, specifically cranial neural crest derived tissues.

#### Differentiation in mesenchymal lineages

**Figure 2:**
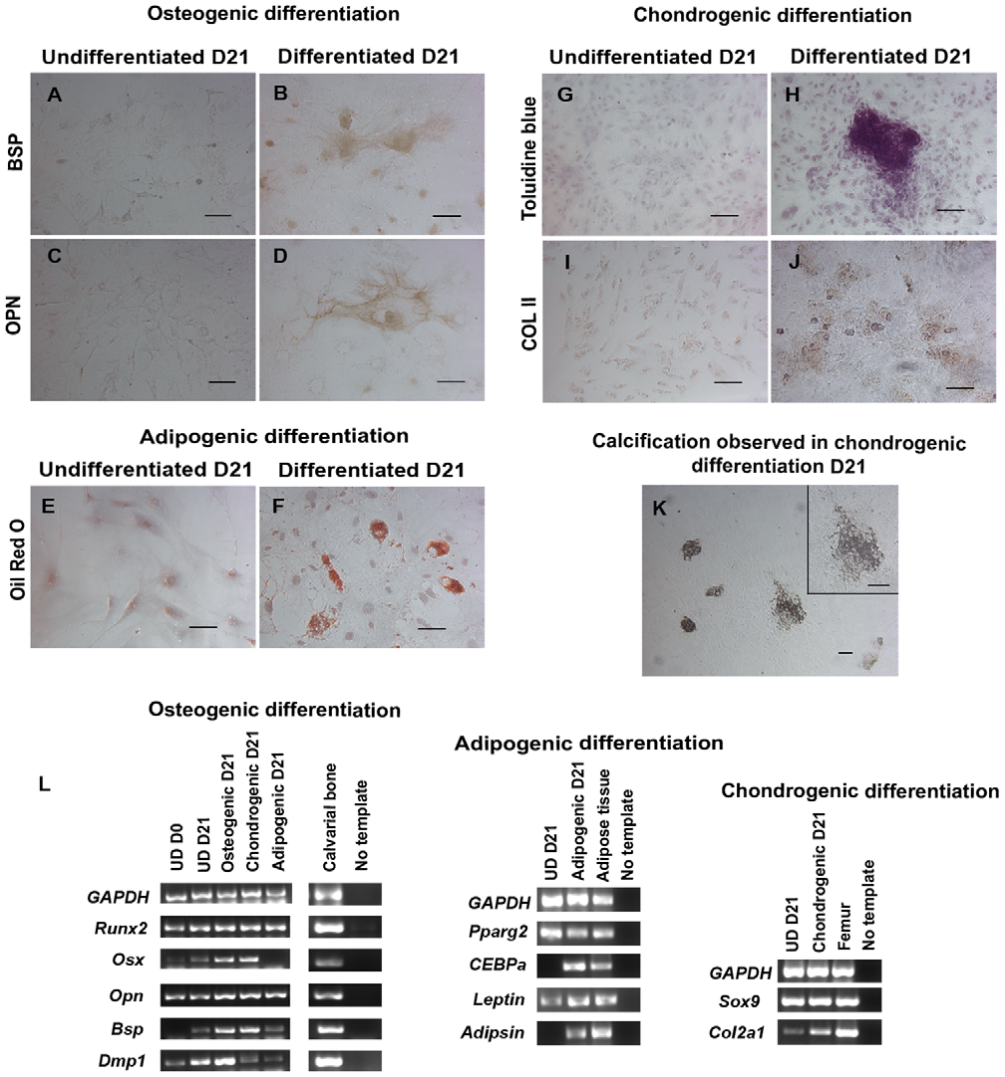
DPSC cultures differentiate into neural crest-derived mesenchymal lineages. (A–D) After 21 days in osteogenic media, differentiated DPSCs were positive for BSP and OPN in a membranous pattern. (F) Cultures in adipogenic media showed lipid droplets-containing cells positive for Oil Red O. (H and J) Cultures in chondrogenic media showed cells in clusters positive for toluidine blue and COL II. (K) Mineralized nodules were observed in cells treated with the chondrogenic media after 21 days. The inset depicts a mineralized nodule in high magnification. (L) RT-PCR confirmed the staining results. For osteogenic differentiation (Osteogenic D21), RT-PCR showed expression of osteoblast- associated genes *Runx2*, *Osx*, *Opn*, *Bsp,* and *Dmp1*. For adipogenic differentiation, induced cells (Adipogenic D21) expressed *Pparg2* and *CEBPa*, adipogenic transcription factors, as well as *Leptin* and *Adipsin,* markers of adipocytes. For chondrogenic differentiation, treated cells (Chondrogenic D21) expressed chondrocyte-associated genes *Sox9* and *Col2a1*. (A, C, E, G, and I) Confluent undifferentiated cells cultured in stem cell media at day 21 (UD D21) expressed some of differentiation genes, but were negative by staining for all differentiation markers and Oil Red O. Cells were counterstained with hematoxylin. RNA isolated from mouse calvarial bone, adipose tissue, and femur was used as positive control for gene expression. Scale bars indicate 100 µm.

DPSCs exposed to osteogenic, adipogenic, and chondrogenic media for 21 days stained positive for proteins, differentiation markers (Figures 2A–2K), and expressed transcripts (Figure 2L) specific to each respective differentiation lineage.

Immunoperoxidase staining of DPSCs cultured in osteogenic media showed cytoplasmic pattern of bone sialoprotein (BSP) and osteopontin (OPN) in differentiated cells, but not in undifferentiated cells (Figures 2A–2D) [28]. RT-PCR showed that differentiated cells expressed osteogenic markers (Figure 2L, Osteogenic differentiation); *runt related transcription factor 2* (*Runx2)*, *osterix* (*Osx*), *Opn, Bsp* and *dentin matrix protein1* (*Dmp1*) [29], [30]. OPN, BSP and DMP1 proteins are present in mineralized tissues such as bone, dentin, and cementum [31], [32]. These results suggest that DPSCs cultured in osteogenic media differentiate into osteoblast/odontoblast-like cells.

Small clusters of lipid-containing cells were first observed on day 7 in cells exposed to adipogenic media (data not shown) [33]. Treated cells showed expression of *peroxisome proliferator-activated receptor gamma* (*Pparg2)* and *CCAAT/enhancer binding protein alpha (CEBPa)*, which are transcription factors for adipocyte differentiation, as well as, *leptin* and *adipsin*, which are specific markers of adipocytes (Figure 2L, Adipogenic differentiation) [33]. Despite *Pparg2* and *leptin* expression in undifferentiated cells, Oil Red O-positive lipid-containing adipocytes were observed only in differentiated cells but not in undifferentiated cells (Figures 2E–2F).

DPSCs cultured in chondrogenic media were first observed to display changes from spindle-shaped cells to clusters of round and cuboidal cells representing chondrocyte-like morphology on day 10 [34], [35]. After 21 days, these cells secreted matrix with high proteoglycan content which stained positive for toluidine blue (Figure 2H) and collagen type II (COL II) (Figure 2J) and formed calcified nodules (Figure 2K), indicating that these cells form cartilage matrix [36]. RT-PCR demonstrated that treated cells in chondrogenic media expressed *Sox9* and increased expression of collagen type II (*Col2a1*) genes which are specific markers for chondrocyte differentiation [37]. We did not observe these chondrogenic hallmarks in undifferentiated cells despite chondrogenic gene expression in such cells (Figure 2L, Chondrogenic differentiation).

RT-PCR showed chondrogenic and adipogenic markers in cells treated with osteogenic media; however, COL II- and/or Oil Red O-positive cells were not observed in this culture (data not shown). Interestingly, we found BSP- and OPN- positive cells, as well as Oil Red O positive-adipocytes in the chondrogenic media (data not shown). This is consistent with previous studies that showed BMP2 is an inducer of not only chondrogenic differentiation but also osteoblastic and adipogenic differentiations [38]. *Osx, Bsp, Dmp1, Pparg2, leptin, Sox9,* and *Col2a1*, were expressed in confluent undifferentiated cells in stem cell media at day 21 (Figure 2L), suggesting these are default differentiation pathways of DPSCs in confluent cultures. Alternatively, it has been previously demonstrated that bone marrow stromal cells display an osteogenic imprinting program and express messengers of these proteins in undifferentiated cells [39].

Intriguingly, Q-RT-PCR showed that *Nanog* and *Klf4* were down-regulated during differentiation of DPSCs (Figure S3), suggesting that like embryonic stem cells, these pluripotent genes play a role in maintaining DPSC stemness and thus are down-regulated during differentiation [40].

#### Differentiation in non-mesenchymal lineages

**Figure 3:**
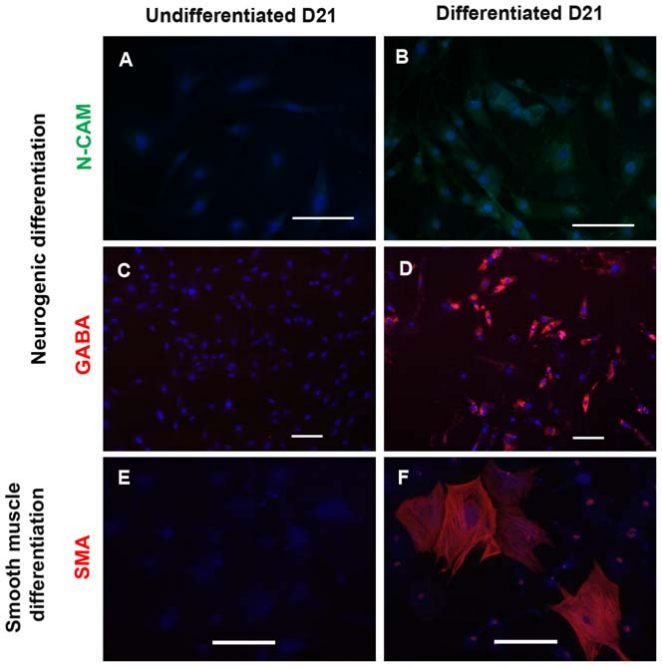
DPSC cultures differentiate into neural crest non-mesenchymal lineages. (B and D) Neuronal-induced cells stained positively for neuronal markers; N-CAM ( in green), and GABA (in red). (F) DPSCs in smooth muscle differentiation media showed smooth muscle-like phenotype that stained positively for smooth muscle actin (in red). (A, C, and E) Undifferentiated cells in stem cell media at day 21 did not stain positively for any of the three differentiation markers. DAPI used for nuclei staining is depicted in blue. Scale bars indicate 100 µm.

After exposing DPSCs to neurogenic media, differentiated cells stained positive for N-CAM, a neuronal adhesion molecule, and γ-aminobutyric acid (GABA), an inhibitory neurotransmitter produced by GABAergic neurons (Figures 3A–3D) [41], [42]. We also treated DPSCs with PDGF-BB to induce smooth muscle differentiation [43]. Following day 21 of PDGF-BB, DPSCs showed smooth muscle-like morphology, and stained positive for α-smooth muscle actin (Figures 3E, 3F). However, we could not observe any positive cells of either neurogenic or smooth muscle differentiations in the undifferentiated cells (Figures 3A, 3C, and 3E).

### DPSCs generate single cell colonies

Colony-forming capacity is a characteristic growth pattern of many adult stem cells including bone marrow mesenchymal stem cells, and has been used as an indicator of self-renewal [44]. Indeed, a single stem cell should create a colony of progeny cells with all or some daughter cells identical to the original cell. Initially, we attempted to demonstrate colony formation from single DPSC by Fluorescent Activated Cell Sorting (FACS) mediated single cell deposition. Unfortunately, this approach was unsuccessful possible due to sensitivity of DPSCs to cell sorting. Thus we utilized clonal rings commonly used to generate single cell colonies from mesenchymal stem cells [45]. We first seeded cells at several dilutions and determined that the limiting dilution to obtain single colonies was 50–100 cells/cm^2^. After 24 hrs adherent single cells were marked and monitored everyday for the formation of colonies. At day 10, we observed 12 colonies in 600 cm^2^ (four 150 cm^2^ petri dishes) (Figures S4A-S4D). After clonal isolation, we cultured these clones in several passages and 5 of 12 survived and proliferated for further characterization.

Like the non-clonal populations, RT-PCR of clones expressed the pluripotency genes, *Klf4* and *Nanog*, and neural crest developmental genes, but not mesodermal developmental genes (Figure S4E). Compared to non-clonal populations, clones definitively expressed higher and consistent levels of *Pax3,* a cardiac neural crest developmental gene [46]. All clones demonstrated strong expression of neural crest and neural precursor genes including *Vimentin* (Figure S4F). *Vimentin* is expressed in mesenchymal cells irrespective of their origin or in cells undergoing epithelial-mesenchymal transition such as migratory neural crest cells [47]. Additionally, the clones showed variable *Dmp1* but not *Dspp*, indicating an undifferentiated state of dental pulp cells.

### In vitro differentiation of DPSC clones

**Figure 4:**
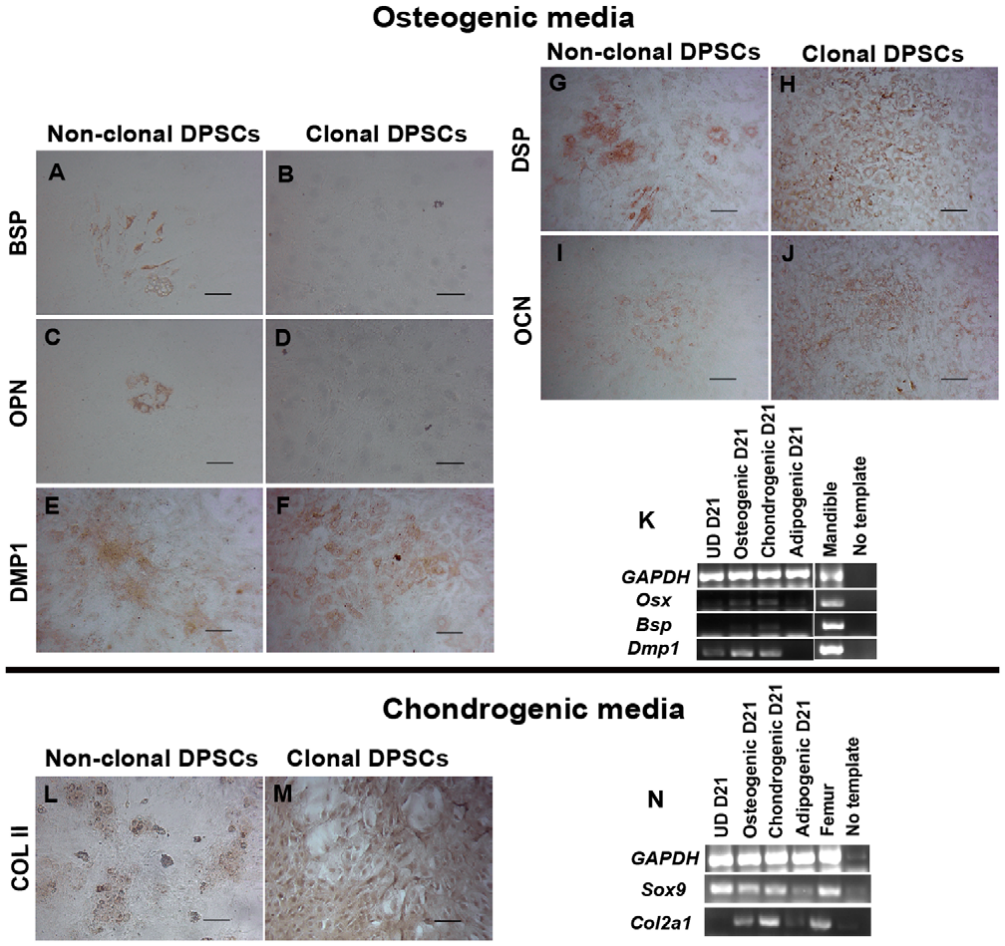
Non-clonal and clonal DPSC cultures differentiate into mesenchymal lineages. Treatment of DPSC clones with osteogenic media resulted in different outcomes compared to the differentiation of non-clonal DPSCs. (A–D) Clones did not produce positive cells for BSP and OPN while non-clonal populations showed clusters of positive cells for these markers. (E–J) Non-clonal and all clonal DPSC cultures in osteogenic media stained positively for DMP1, DSP, and OCN. (K) Corresponding to staining results, RT-PCR shows the expression of differentiation genes following treatment of clones with osteogenic media, lack of *Bsp*, but strong expression of *Dmp1* was observed in the differentiation of DPSC clones (Osteogenic D21). (L and M) Non-clonal and 4 out of 5 clones (C5–C8) stained positively for COL II after treatment with chondrogenic media. (N) RT-PCR shows strong expression of chondrogenic markers, *Sox9* and *Col2a1*, in DPSCs cultured in chondrogenic media (Chondrogenic D21). Staining and RT-PCR are all derived from a representative clone (C6). RNA isolated from mouse mandible and femur was used as positive control for gene expression. Scale bars indicate 100 µm.

To determine if DPSC clones show multi-differentiation in neural crest lineages, we performed *in vitro* differentiation by exposing 5 clones to the same differentiating media used to differentiate the non-clonal populations. Prior to differentiation, we analyzed Q-RT-PCR levels for *Klf4* and *Nanog* compared to non-clonal populations and dental pulp tissue (Figure S5). In turn, *Nanog* and *Klf4* expression were variable among the clones but comparable to the non-clonal populations. Thus, we assumed that clonal populations should exhibit same differentiation capacity and fate as compared to non-clonal populations (Figures 4A–4J, 4L and 4M).

Unlike differentiation of non-clonal cultures (Figures 4A, 4C), we did not observe any differentiated clonal cells positive for BSP and OPN in osteogenic media (Figures. 4B, 4D), corresponding to low expression of *Bsp* in differentiated clones (Figure 4K). BSP is the osteoblast-specific protein found in bone matrix and highly secreted by osteoblasts [48]. In contrast, consistent with *Dmp1* expression (Figure 4K), non-clonal and all clones in osteogenic media stained positive for DMP1 (Figures 4E, 4F), dentin sialoprotein (DSP) (Figures 4G, 4H) and osteocalcin (OCN) (Figures 4I, 4J); all of which are non-collagenous proteins secreted by odontoblasts and found in dentin matrix [31], [49], [50]. DSP is particularly considered a dentin-specific protein which is highly expressed by odontoblasts [48].These results suggest the presence of odontoblast-like cells more abundant in clonal differentiations. This osteogenic media has been used to differentiate odontoblast-like cells from dental pulp cells in previous studies, thus it is not surprising that DPSCs can differentiate into odontoblast-like cells when induced with such media [51]. However, the clonal populations showed a more odontogenic than osteogenic phenotype under this condition. Furthermore, all non-clonal DPSCs and 4 out of 5 DPSC clones stained positively for COL II (Figures 4L, 4M) after treatment with chondrogenic media, which was confirmed by the *Sox9* and *Col2a1* expression (Figure 4N).

**Figure 5:**
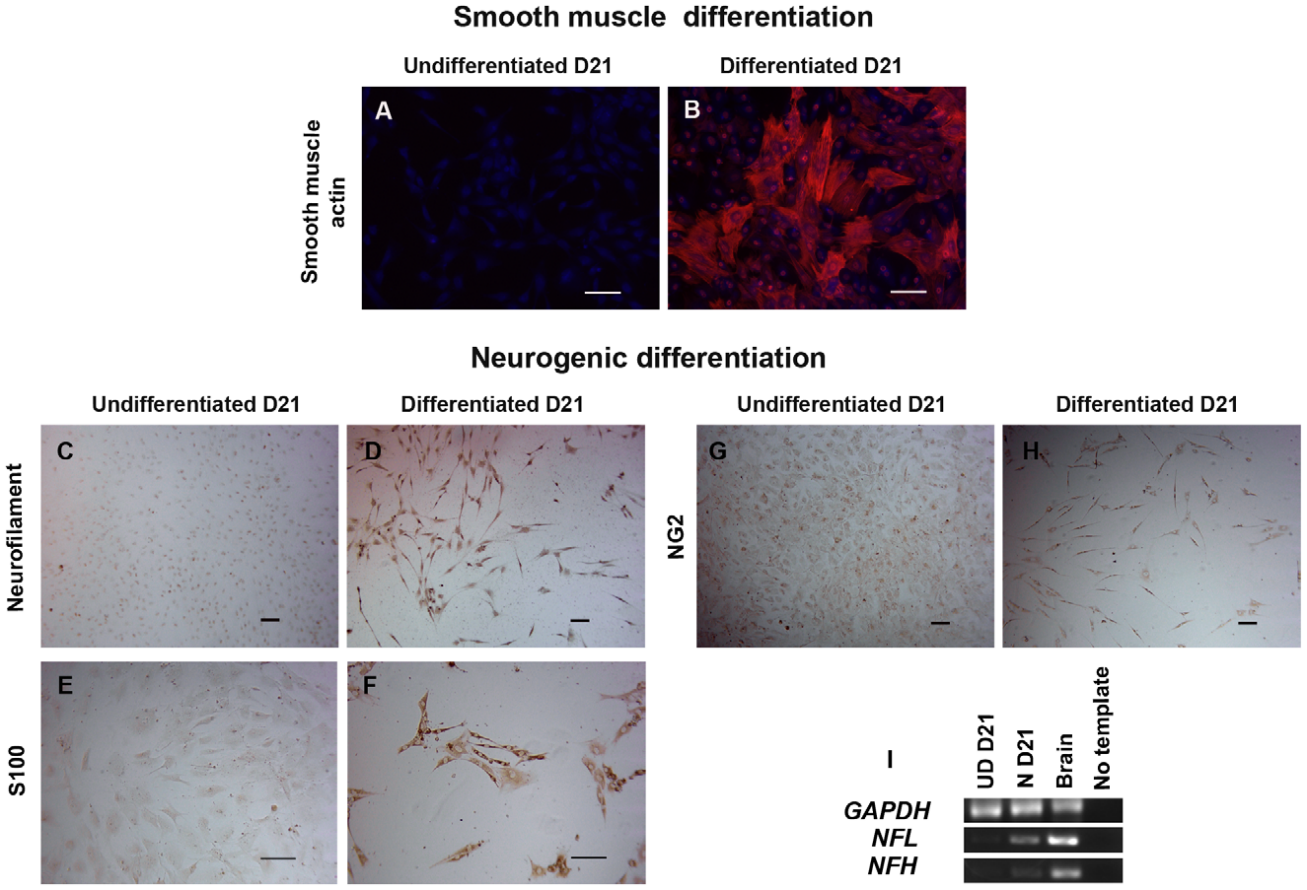
DPSC clones differentiate into neural crest non-mesenchymal lineages. (B) All clones showed smooth muscle-like cells positive for smooth muscle actin (in red). (D, F, and H) Three clones (C6–C8) showed positive cells for neurofilament (NF-160/200), S100, and NG2. (I) The NF staining was confirmed by RT-PCR of neurofilament-light (NFL) and -heavy (NFH). (A, C, E, and G) Undifferentiated clones were negative for smooth muscle and neuronal markers. DAPI used for nuclei staining is depicted in blue. Scale bars indicate 100 µm.

In contrast, all non-clonal and clonal DPSC cultures acquired a smooth muscle-like morphology and stained positive for α-smooth muscle actin after treatment with PDGF-BB media (Figures 3F, 5B). In addition, Q-RT-PCR of smooth muscle genes showed higher expression of smooth muscle- and pericyte-related genes (*Sm22-alpha*, *Sma*, *SMHC*, and *calponin*) in the undifferentiated clonal populations as compared to non- clonal (Figure S6). In turn, the differentiated progeny derived from the clonal populations showed a pattern of smooth muscle maturation with significantly increased levels of *myocardin, Sm22-alpha, Sma*, *SMHC*, and *calponin* (>10 folds higher than non-clonal differentiated cells and >100 folds higher compared to smooth muscle cells) whereas in the non-clonal populations this trend of maturation is not apparent (Figure S6) [52], [53]. Three out of five clones stained positive for neurofilament (NF-160/200) (Figure 5D), S100 (Figure 5F), and NG2 (Figure 5H) which are neuronal cytoskeletal, glial, and oligodendrocyte markers, respectively, after induction with neurogenic media [54]. The NF staining was confirmed with RT-PCR of neurofilament-light (NFL) and -heavy (NFH) (Figure 5I). In conclusion, 3 out 5 clones could differentiate into odontoblast-like, chondrocyte-like, smooth muscle-like and neuronal-like cells, demonstrating their neural crest stem cell capacity (comparison summarized in Table S1). Nevertheless, each clone showed some variability in lineage differentiation capacity and efficiency, which reflects heterogeneity and/or a complex hierarchy of progenitor/stem cells in our cultures.

### DPSCs produce dentin-like structure in vivo

To determine *in vivo* differentiation of DPSCs, we transplanted DPSCs labeled with a red fluorescence dye (PKH-26) intramuscularly and subcutaneously in immune compromised *Rag1* null mice to avoid immune rejection [55]. We used murine bone marrow stromal cells (BMSCs) cultured and transplanted under the same conditions in order to compare DPSCs *in vivo* differentiation capacity with another mesenchymal line derived from a different origin (mesodermal).

Two-weeks after intramuscular (IM) transplantation, non-clonal DPSCs identified as PKH-26+ (Figure S7A), created compacted collagen bundles as indicated by Masson's trichrome (Figure S7B). The presence of collagen fibers was confirmed with polarized light microscopy (Figure S7C). Staining for dentin or bone proteins was only slightly positive (data not shown), suggesting that the compacted collagen bundles did not resemble dentin- or bone-like structure. We then hypothesized that longer *in vivo* transplantation will result in more mature phenotype of the transplanted cells. Nonetheless, 12-week IM transplantations of non-clonal, clonal DPSCs, and BMSCs, only generated abundant immature collagen fibers that stained blue in Masson's trichrome (Figures S7D-S7F), and polarized light (Figures S7G-S7I) but were negative for dentin or bone proteins (data not shown). This indicates that the skeletal muscle is not an inductive environment for formation of mature mineralized matrix.

To explore if DPSCs can form more mature matrix structures *in vivo*, we used a more permissive model of matrix formation, subcutaneous (SC) transplantation with hydroxyapatite/tricalcium phosphate scaffolds (HAp/TCP) [2]. Here we transplanted three clones separately, which expressed high levels of *Nanog* and *Klf4* by Q-RT-PCR (Figure S5), and showed multi-lineage neural crest differentiation capacity (Table S1). In these transplants we also used BMSCs as control cell line.

**Figure 6:**
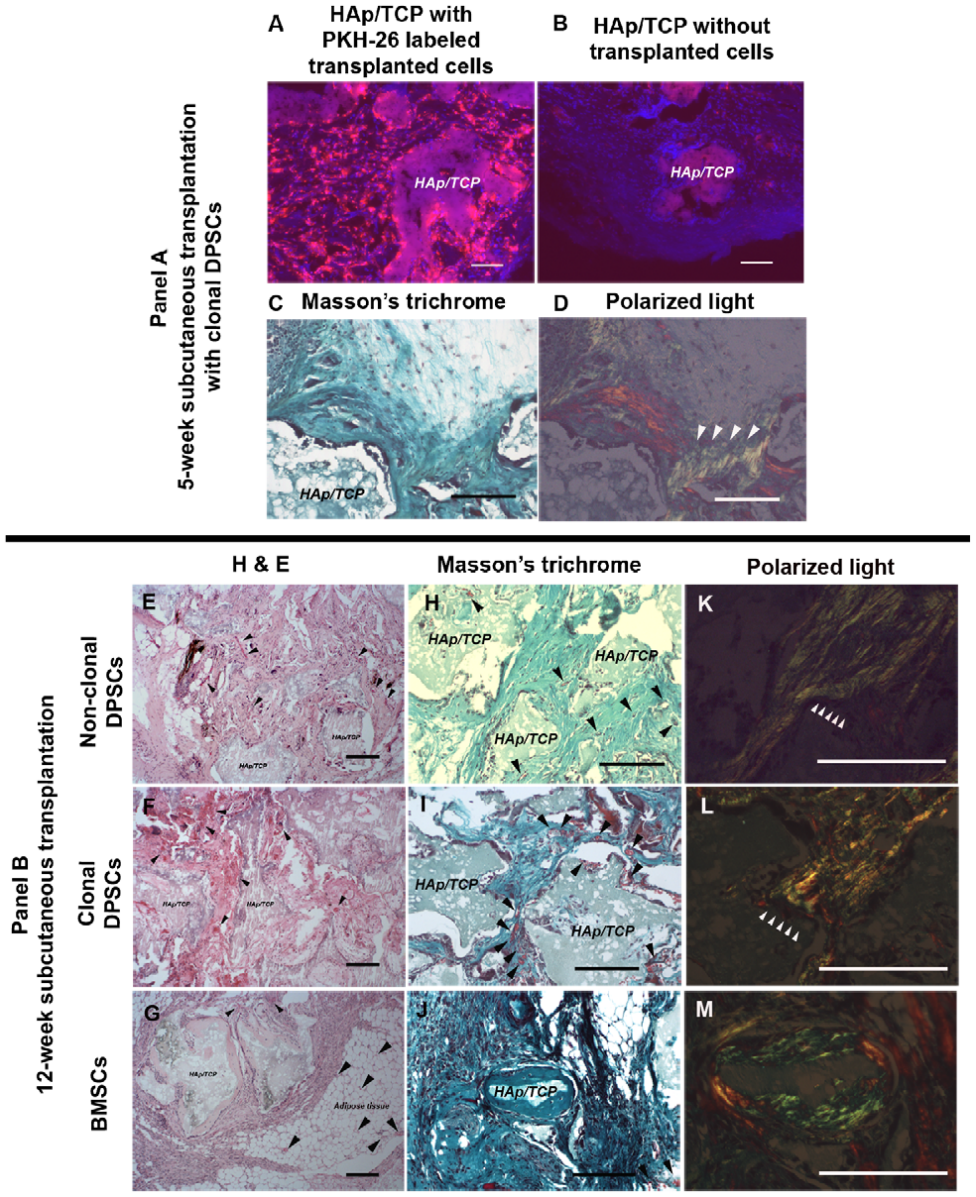
Subcutaneous transplantation of DPSCs and BMSCs. Non-clonal and clonal DPSCs (C6–C8) were labeled with PKH-26 before transplantation to track cells *in vivo*. Panel A shows 5-week subcutaneous (SC) transplantation of DPSC clones with HAp/TCP. (A and B) PKH-26+ cells SC transplanted with HAp/TCP were identified in bright red fluorescent color whereas the HAp/TCP scaffold without cells was devoid of red fluorescent cells despite some autofluoresence derived from HAp/TCP. The HAp/TCP scaffold without cells did not show mineralized tissues (data not shown). Most of transplanted cells surrounded HAp/TCP. (C) These clonal cells generated collagen-forming matrix shown in blue of Masson's trichrome. (D, white arrowheads) Polarized light was used to confirm collagen formation. Panel B shows 12-week SC transplantation of non-clonal DPSCs, clonal DPSCs, and BMSCs with HAp/TCP. (E–G) H&E staining showed the morphology of tissues created by non-clonal, clonal DPSCs and BMSCs. Capillaries and small blood vessels near mineralized matrices were found in DPSC transplants. In contrast, BMSCs formed bone condensations near HAp/TCP devoid of vessels. In BMSC transplants capillaries were found mainly in the adipose tissue. The black arrowheads indicate the location of capillaries and small blood vessels. (H–J) Masson's trichrome showed the intensive staining of collagenous extracellular matrices generated by both DPSCs and BMSCs. (K–M) polarized light images with high magnification of transplanted tissues showed different collagen arrangement produced by DPSCs and BMSCs. (K and L, white arrowheads, and M) The white arrowheads indicate the direction of polarized collagen fibers created by DPSCs run perpendicularly to HAp/TCP while that created by BMSCs do not run perpendicularly to scaffold surface. DAPI used for nuclei staining is depicted in blue. *HAp/TCP*  =  Hydroxyapatite/tricalcium phosphate. Scale bars indicate 200 µm.

**Figure 7:**
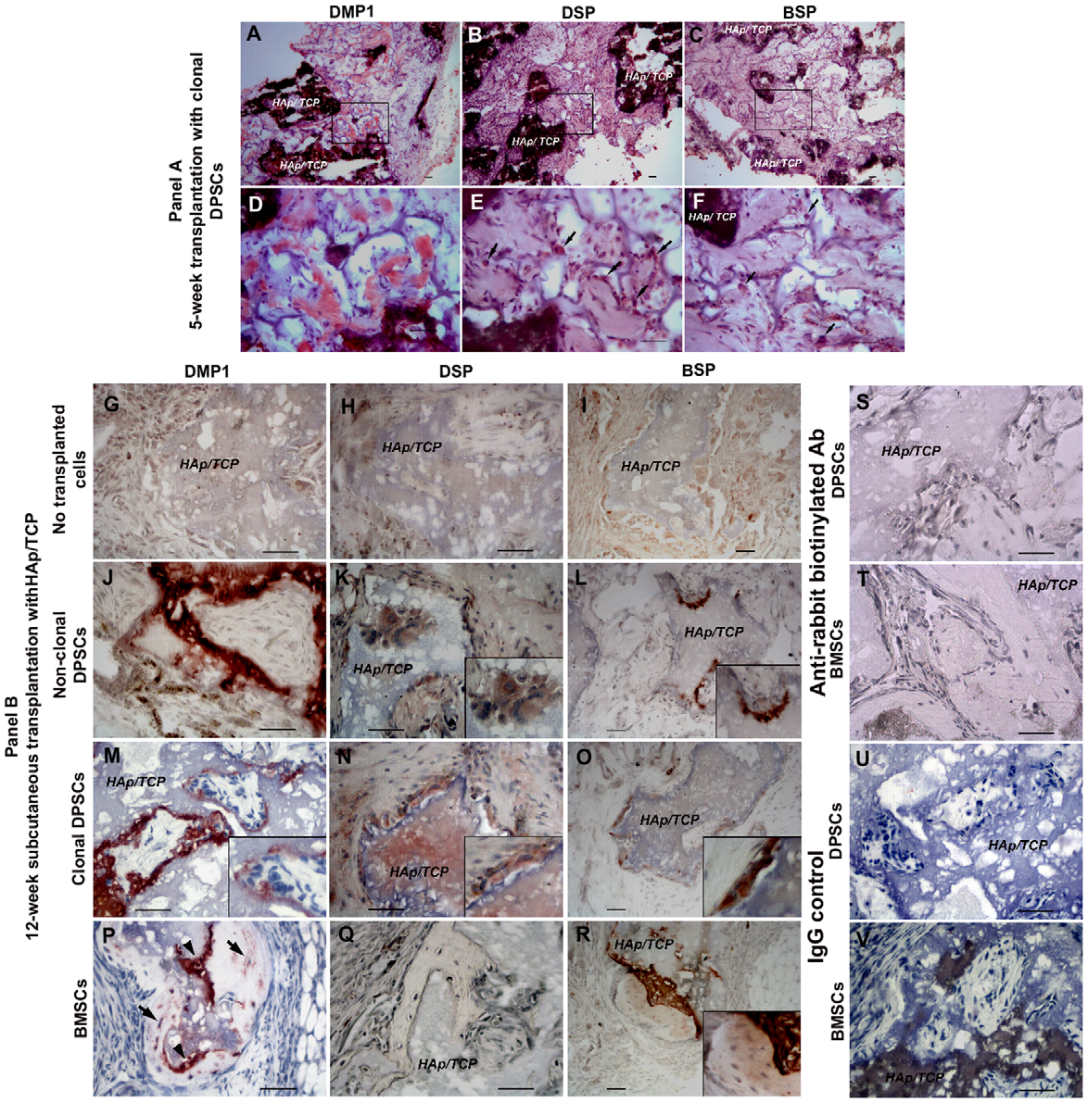
Immunohistochemistry of subcutaneous DPSC and BMSC transplanted tissues. Panel A shows the staining of 5-week subcutaneous (SC) clonal DPSC transplanted tissues. (A–C) Three DPSC clones (C6–C8) secreted extracellular matrices which were positive for DMP1, slightly positive for DSP and BSP by immunoperoxidase staining. (D–F) High magnification images correspond to the rectangular areas of A–C. (D) Strong DMP1 staining was seen in the matrices. (E and F, arrows) Strong positive staining for DSP and BSP were observed in the transplanted cells. Panel B shows the staining of 12-week SC transplantations of non-clonal DPSCs, clonal DPSCs, and BMSCs with HAp/TCP. (G–I) Only HAp/TCP transplantation without donor cells did not show any extracellular matrices positive for dentin and bone proteins. (J–O) Both non-clonal and clonal DPSCs secreted extracellular matrices which were strongly positive, but different intensity, to DMP1, DSP, and BSP. All insets in each figure show high magnification images of the transplanted tissues to illustrate the morphology and positive intracellular staining of transplanted cells. (K, M, and N insets) transplanted cells were elongated, polarized, and showed very close relation to scaffold surface, resembling odontoblast-like cell morphology. (K, L, M, N, and O insets) Positive staining of DMP1, DSP, and BSP was seen in transplanted DPSCs. Some cells showed elongation and polarization. (P, arrowheads and arrows) BMSC transplanted cells showed osteoblast-like cells (arrowheads) lining bone surface, and osteocyte-like cells (arrows) in lacunae which stained positively for DMP1. (Q) Negative staining for DSP was observed in bone matrix and transplanted BMSC cells. (R, inset) Positive staining for BSP was observed in bone matrix secreted by transplanted BMSCs. (S–V) Transplanted sections stained with only anti-rabbit biotinylated antibody or IgG isotype were used as negative control. *HAp/TCP*  =  Hydroxyapatite/tricalcium phosphate. Scale bars indicate 200 µm.

Five weeks after SC transplantation with HAp/TCP, PKH26-labeled DPSC clones (Figure 6A) generated collagen forming-tissue demonstrated by Masson trichrome (Figure 6C) and polarized light (Figure 6D, white arrowheads). In addition, PKH-26+ clonal cells produced extracellular matrices which showed strong staining for DMP1 (Figures 7A, 7D), and slightly positive staining for DSP (Figures 7B, 7E) and BSP (Figures 7C, 7F). However, strong positive staining of DSP (Figure 7E, black arrowheads) and BSP (Figure 7F, black arrowheads) was observed in the cytoplasm of transplanted cells. DPSC clones were negative for dentin and bone proteins prior to transplantation. All antibodies for dentin and bone matrices showed appropriate staining pattern in tooth sections used as positive control to confirm the antibody specificity (Figure S8). We observed that these mineralized tissues formed by DPSCs contained abundant microvessels that stained positive for CD31, an endothelial marker (Figure S9A). We consistently observed some PKH-26+ (donor DPSCs) cells adjacent to these microvessels. These PKH-26+ cells seem scattered around vessels. Staining for α-smooth muscle actin revealed that these microvessels did not contain a thick muscle layer but were wrapped by pericyte-like cells that were SMA positive and some were also PKH-26+ (Figures S9C, S9D, S9F). Many of these microvessels seem fenestrated and stained positive for VEGF receptor 3 (Figures S9E, S9F) [56].

Interestingly, the 12-week SC transplantation showed different composition of mineralized matrices depending on the donor cells, DPSCs and BMSCs. The H&E and Masson's trichrome showed bone-like structure created by BMSCs (Figures 6G, 6J), but not in non-clonal and clonal DPSC transplantations (Figures 6E, 6F, 6H, and 6I). The structures produced by both DPSC populations demonstrated collagen-forming matrices in many areas that were arranged perpendicularly to the surface, which was confirmed using polarized light (Figures 6K, 6L). Corresponding to previous studies, the DPSC transplantation showed that elongated cells created collagen fibers which run perpendicularly to HAp/TCP surfaces, resembling the morphology and arrangement of odontoblasts (Figures 6K, 6L, white arrowheads) [2], [3], [50]. In contrast, in the BMSC transplantation we observed osteocyte-like cells trapped in matrix of collagen fibers which did not run perpendicularly to the mineralized matrix (Figures 6J, 6M) [2], [3].

As negative control, the SC transplantation with only HAp/TCP, did not show any positive staining of DMP1, DSP, and BSP, respectively (Figures 7G–7I). Conversely, immunohistochemistry in DPSC transplants showed positive DMP1, DSP, and BSP staining in both cytoplasmic area and extracellular matrices (Figures 7J–7O). We observed elongated and polarized cells that were positive for DMP1 and DSP in the DPSC transplantations (Figures 7K, 7M, 7N insets). On the other hand, the bone-like structures created by BMSCs were negative for DSP, but positively for BSP (Figures 7Q and 7R inset). We also observed positive DMP1 staining in both DPSC-derived odontoblast-like cells and BMSC-derived osteoblast-/osteocyte-like cells (Figures 7M inset, 7P osteoblast-like cells indicated by arrowheads, osteocyte-like cells indicated by arrows). These results, based on tissue morphology and antibody staining, indicate that *in vivo* DPSCs differentiated into odontoblast-like cells and generated dentin-like matrix whereas BMSCs differentiated into osteoblast-like cells and formed bone condensations.

**Figure 8:**
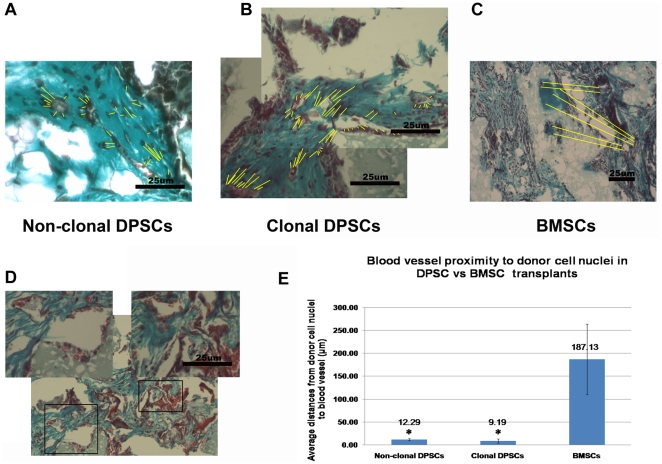
Proximity of donor cell nuclei to blood vessels in DPSC and BMSC transplantation. The average distance of donor nuclei in condensed matrices to nearest capillaries was measured using ImageJ v1.43u (Wayne Rasband, NIH; http://rsb.info.nih.gov/ij). The distances between donor nuclei to blood vessels were represented by yellow lines. (A and B) In the 12-week DPSC transplants, abundant microvessels with fenestrated morphology were seen in close proximity to odontoblast-like cells in both non clonal and clonal DPSC transplants. (C) In BMSC transplants microvessels were predominantly found in areas rich in adipocytes whereas mineralized areas formed by BMSCs were distant to microvessels. Microvessels were mature as circulating red blood cells can be seen throughout in both DPSC and BMSC transplants. (D) Abundant microvessels with fenestrated morphology containing circulating red blood cells (insets) are seen in close proximity to mineralized matrix formed by DPSCs. (E) The bar graph shows that the average distance from odontoblast nuclei to nearest capillary was significantly close (9 µm ± 4.49) and consistent across all DPSC transplants whereas the average distance of osteocyte nuclei in bone condensations to nearest microvessels were significantly farther (187 µm ± 76.88). The average distances from donor cell nuclei to blood vessels in non-clonal DPSCs (n = 5, n; numbers of measured area) or clonal DPSCs (n = 7) and BMSCs (n = 5) are statistically significant. Student's t-test calculated * P≤0.05. Error bars represent ±SEM. Scale bars indicate 25 µm.

Upon closer examination of the odontoblastic niche in the 12-week DPSC transplants, similar to 5-week transplants, we observed abundant microvessels in close proximity to odontoblast-like cells with fenestrated morphology surrounded by PKH-26+ cells, in both non clonal and clonal DPSC transplants (Figures 6E, 6F black arrowheads, S9G, 8A, and 8B). In contrast, matrices that were generated by BMSC transplants surrounded areas rich in HAp/TCP (Figures 6G black arrowheads, and 6J). Although microvessels were observed in the BMSC transplants, the BMSC mineralized areas did not show close proximity to vessels (Figure 8C black arrowheads). Interestingly, in the BMSC transplants microvessels were predominantly found in areas rich in adipocytes (Figures 6G and S9H). In both transplants, microvessels were mature as circulating red blood cells can be seen throughout (Figure 8D). Therefore, we performed a quantification of the average distance of donor nuclei in condensed matrices to nearest capillaries. Surprisingly, the distance from odontoblast nuclei to nearest capillary was very close (9 µm +/− 4.49) and very consistent across all DPSC transplants whereas the distance of osteocyte nuclei to capillaries in bone condensations were significantly farther (187 µm+/− 76.88) (Figure 8E). This capillary arrangement resembles dentinogenesis and contrasts early osteogenesis. In dentinogenesis, capillaries are in close proximity to odontoblasts and many of these capillaries are fenestrated whereas in osteogenesis the initial bone condensations occur in avascular areas [57]–[59].

### *Wnt1-Cre/R26R-LacZ* mice demonstrate that DPSCs are neural crest-derived

Although we have shown that non-clonal and clonal DPSCs express neural crest genes and differentiate into neural crest-lineages *in vitro* and form dentin like structures *in vivo*, the developmental origin of our cultured cells collectively called DPSCs could not be distinguished. Consequently, we used a genetic lineage tracing model, the *Wnt1-Cre/R26R-LacZ* mouse, to demonstrate the contribution of neural crest to stem cells in dental pulp [9].

**Figure 9:**
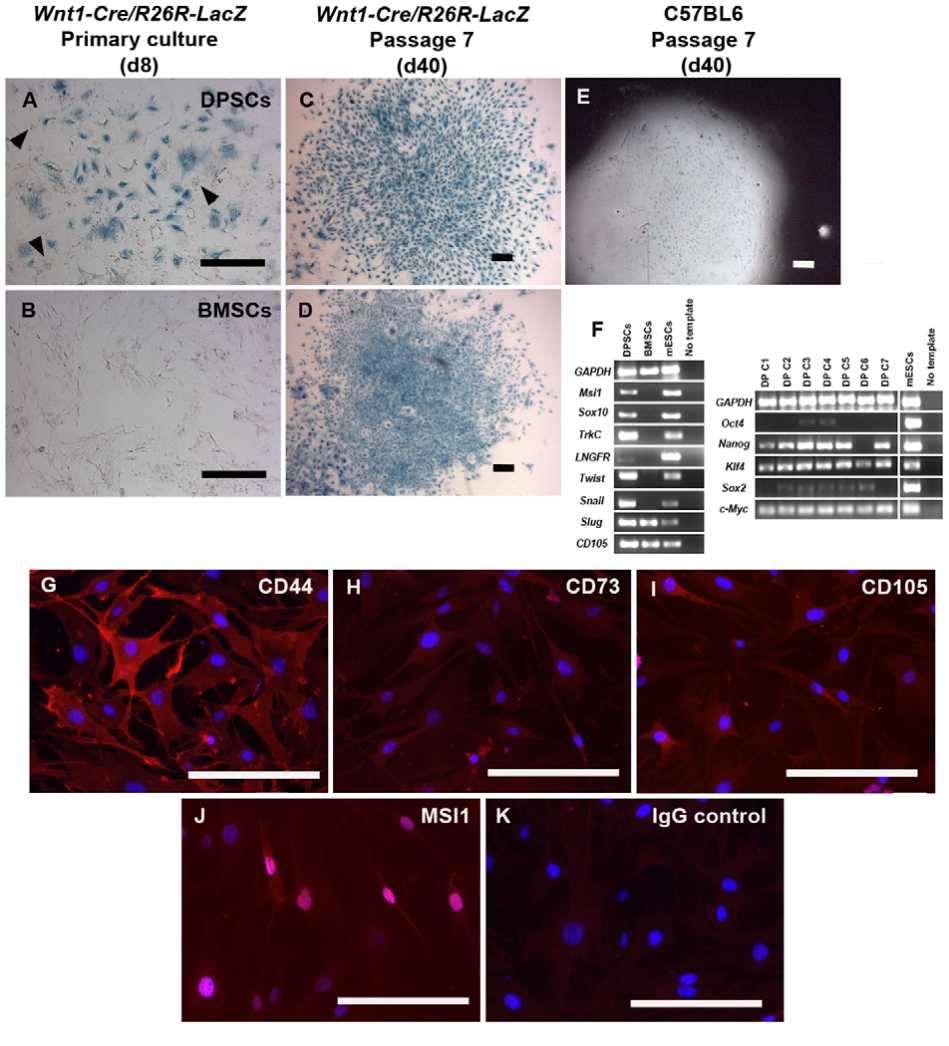
DPSC clones from *Wnt1-Cre/R26R-LacZ* mice. (A) DPSCs first isolated from 4–10 day-old *Wnt1-Cre/R26R-LacZ* showed a majority of *Wnt1*-marked cells in primary culture on day 8 (d8) which stained positively for β-galactosidase with X-gal. A significant minority (10%) of negative cells was also observed in early cultures (arrowheads). (B) Bone marrow stromal cells (BMSCs) isolated from the same *Wnt1-Cre/R26R-LacZ* cultured in the same condition for 8 days showed negative staining for X-gal. DPSCs were successfully cloned on day 7–10. Following several passages until day 40 (d40), all clones generated in this experiment (n = 10) were 100% positive for X-gal. (C and D) Two different *Wnt1* marked clones were shown. (E) A representative DPSC clone from C57BL6 was negative for X-gal. (F) RT-PCR demonstrated that DPSC clones strongly expressed most of neural crest-related genes, *Msi1, Sox10, TrkC, Twist, Snail,* and slightly expressed *LNGFR*, but not in BMSCs. Both DPSCs and BMSCs expressed *CD105*, a known mesenchymal stem cell marker. DPSC clones also expressed pluripotent stem cell genes, *Nanog, Klf4, Sox2* and *c-Myc.* (G–J) Immunofluorescence showed positive staining of mesenchymal stem cell markers, CD44, CD73, CD105, and neural crest-related marker, MSI1 in DPSC clones (in red). (K) Staining with IgG isotype was used as negative control. DAPI used for nuclei staining is depicted in blue. Scale bars indicate 50 µm.

In the *Wnt1-Cre/R26R-LacZ* double-transgenic mice all neural crest derived tissues express reporter driven β-galactosidase. DPSCs isolated from the *Wnt1-Cre/R26R-LacZ* neonatal mice showed a majority of *Wnt1*-marked cells in culture stained positive for β-galactosidase with X-gal. Nonetheless, early in culture a minority of cells (approximately 10%) stained negative for X-gal (Figure 9A, arrowheads). Although we could not confirm the origin of this minority, the percentage of β-galactosidase negative cells is consistent with the highest *Cre/lox* efficiencies reported for other transgenic models [60], [61]. To confirm the neural crest origin of DPSCs, we also isolated bone marrow stromal cells (BMSCs) from the same *Wnt1-Cre/R26R-LacZ* mice and cultured in the same condition. Unlike DPSCs, primary culture of BMSCs at day 8 negatively stained for X-gal, indicating their non-neural crest origin (Figure 9B). A previous study using the same transgenic mouse model, *Wnt1-Cre/R26R-LacZ,* reported that BMSCs did not derive from neural crest [62]. DPSCs were successfully cloned on day 7–10 before the first passage. All DPSC clones (n = 10) stained 100% positive with X-gal (Figures 9C, 9D) and were grown for four passages. Since the majority of cells early in culture stained positive with X-gal, it is unlikely that the homogeneous expression of β-galactosidase in clones derived prior to the first passage resulted from epigenetic changes induced by long-term culture. X-gal staining of C57BL6-derived DPSCs in a late passage was negative (Figure 9E) demonstrating specificity of X-gal staining and ruling out non-specific or mammalian endogenous β-galactosidase activity in the X-gal staining. Like our previous DPSCs, RT-PCR showed that *Wnt1-Cre/R26R-LacZ* derived DPSC clones expressed pluripotent stem cell genes, *Nanog, Klf4, Sox2*, and *c-Myc,* but not *Oct4*. As expected, DPSCs expressed neural crest-related genes, *Msi1, Sox10, TrkC, LNGFR, Twist,* and *Snail,* whereas BMSCs did not express these genes. *Twist*, *Snail*, and *Slug* are expressed by cells in eptithelial-mesenchymal transition (EMT) process during embryological development such as migratory neural crest cells [63]. Previous studies have shown that those EMT-related genes can be expressed by mesenchymal stem cells from bone marrow [64], [65]. Both DPSCs and BMSCs also expressed CD105, also known as endoglin, which is a well-known mesenchymal stem cell (MSC) marker (Figure 9F) [66]. In addition, immunofluorescence showed that DPSC clones in our culture condition stained positively for CD44, CD73, CD105, three MSC markers previously reported in human DPSCs, and stained positively for MSI1, a neural crest-related marker (Figures 9G–9K) [67]–[69] .

**Figure 10:**
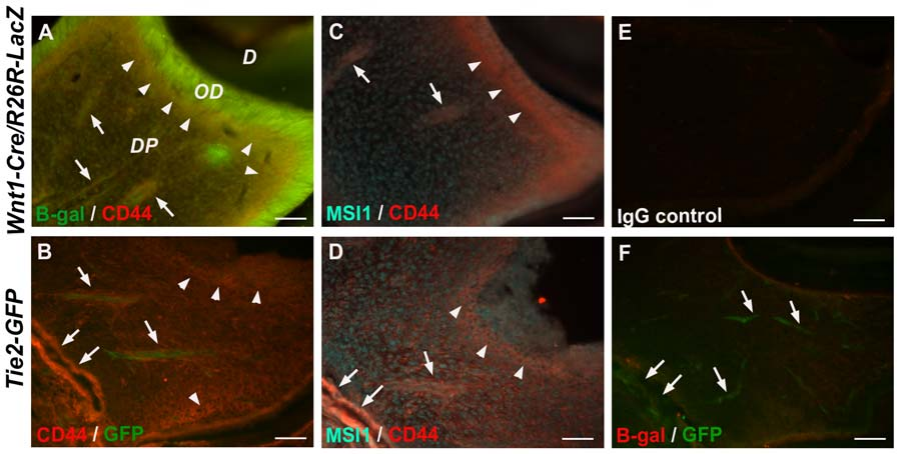
Localization of neural crest-derived stem cells in dental pulp of neonatal mice. (A) Immunofluorescence showed *Wnt1-Cre/R26R-LacZ* derived dental pulp cells and odontoblasts expressing β-galactosidase (B-gal, in green). The co-expression of β-galactosidase and mesenchymal stem cell marker, CD44, in plasma membrane (in red) was observed in DPSCs located in sub-odontoblastic (arrowheads) and perivascular regions (arrows). (B) In *Tie2-GFP* dental pulp only vessels express green fluorescent protein (GFP), CD44 positive cells were particularly located in sub-odontoblastic (arrowheads) and perivascular areas (arrows). (C and D) The co-localization of CD44 (in red) and MSI1 (in pseudo-color cyan), a neural crest-related marker, was observed in cells located in sub-odontoblastic (arrowheads) and perivascular areas (arrows) of both *Wnt1-Cre/R26R-LacZ* and *Tie2-GFP*, respectively. (E) Staining with IgG isotypes are shown as negative control. (F) *Tie2-GFP* derived dental pulp cells did not express β-galactosidase, but blood vessels in this transgenic mouse were labeled by GFP (arrows). *D*  =  Dentin, *DP*  =  Dental pulp, *OD*  =  Odontoblasts. Scale bars indicate 100 µm.

We corroborated the presence of neural crest-derived MSCs in dental pulp of neonatal mice, *Wnt1-Cre/R26R-LacZ* and *Tie2-GFP* that co-expressed MSC and neural crest-related markers. Since neural crest-derived cells of the *Wnt1-Cre/R26R-LacZ* express β-galactosidase under *Wnt1* promoter driven, we studied the expression of MSC markers in *Wnt1* marked cells in dental pulp. From all three of MSC markers expressed by cultured DPSCs (CD44, CD73, and CD105), only CD44 is strongly expressed in dental pulp tissue. Staining of tooth sections with anti-β-galactosidase showed that many dental pulp cells and all odontoblast cells in the *Wnt1-Cre/R26R-LacZ* mouse were positive for β-galactosidase, confirming their neural crest origin (Figure 10A). Cells co-expressing β-galactosidase and CD44 were found in the *Wnt1-Cre/R26R-LacZ* dental pulp particularly in the sub-odontoblastic and perivascular regions, indicating DPSCs are of neural crest origin (Figure 10A, arrowheads and arrows, respectively). *Tie2-GFP* derived dental pulp showed CD44-positive cells in the same areas found in the *Wnt1-Cre/R26R-LacZ* (Figure 10B, arrowheads and arrows). The CD44 staining of both transgenic mice's teeth indicates DPSCs are preferentially localized in perivascular niches as suggested in previous studies [70]–[72].

Additionally, we used another double staining of MSI1 and CD44 to confirm co-expression of CD44, a mesenchymal stem cell marker, and MS1, a neural crest-related marker, by DPSCs. Like the co-staining of β-galactosidase and CD44, DPSCs stained positively for MSI1 and CD44 preferentially located in the same areas; underneath odontoblast cell layer and surrounding blood vessels (Figures 10C, 10D, arrowheads and arrows, respectively). As expected, we did not observe any positive cells for β-galactosidase in the *Tie2-GFP* derived dental pulp and IgG isotype control staining (Figures 10E, 10F). Accordingly, we observed blood vessels in the *Tie2-GFP* dental pulp expressing GFP (Figures 10B, 10F, arrowheads). The co-expression of β-galactosidase or neural crest-related and MSC markers indicates the existence of neural crest-derived DPSCs in murine neonatal teeth.

**Figure 11:**
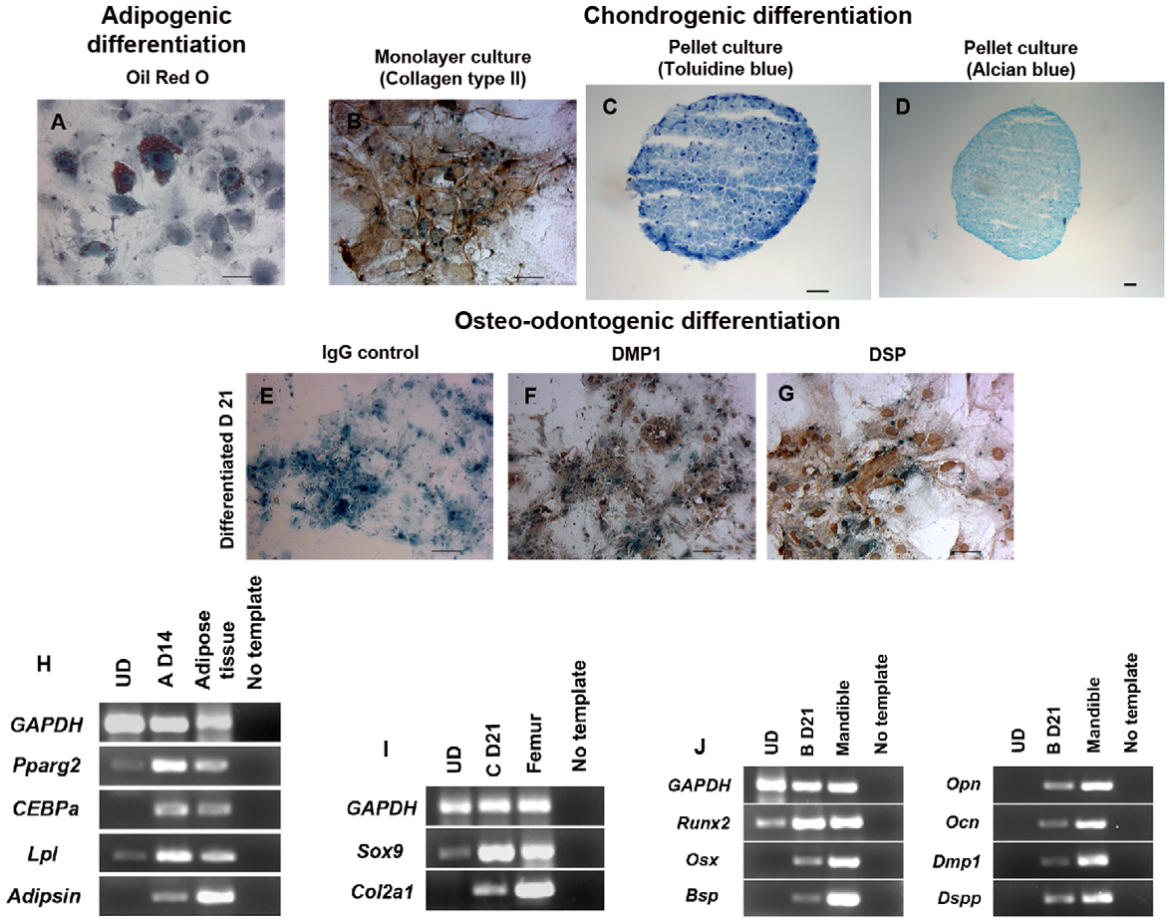
Multi-differentiation capacity in neural crest-derived mesenchymal lineages of *Wnt1*-marked DPSCs. (A) Double staining showed that X-gal+ treated cells in adipogenic media demonstrated lipid droplets-containing cells positive for Oil Red O. (B) Chondrogenic differentiation by monolayer method showed that a cluster of chondrocyte-like cells derived from DPSCs secreted extracellular matrices positive for COLII. (C and D) DPSCs cultured in chondrogenic media by pellet method formed aggregates containing highly proteoglycan which stained positively for toluidine blue and alcian blue, respectively. (E) Differentiated DPSCs in osteo-odontogenic media stained with IgG isotype as negative control showed X-gal positive staining. (F and G) DPSCs treated in osteo-odontogenic media for 21 days secreted dentin matrices stained positively for DMP1 and DSP; those extracellular matrices were not found in cells cultured in stem cell media. (H–J) RT-PCR from each differentiation confirmed the staining results. DPSCs treated in each differentiation media up-regulated specific adipogenic (A D14), chondrogenic (C D21), and osteo-odontogenic (B D21) genes after 14 to 21-day culture. RNA of mouse adipose tissue, femur, and mandible was used as positive control while the reaction without cDNA was used as negative control. Scale bars indicate 100 µm.

Next, we determined *in vitro* multi-differentiation capacity in neural crest lineages of the *Wnt1-Cre/R26R-LacZ* derived clonal DPSCs using the same differentiation protocols. Cells treated with adipogenic media generated lipid-containing cells positive for Oil Red O (Figure 11A). We performed chondrocyte differentiation in both monolayer and cell pellet methods cultured in chondrogenic media [73], [74]. After 21 days differentiated cells in the monolayer secreted extracellular matrix stained positively for COLII (Figure 11B). In addition, the chondrogenic pellet culture conditions induced DPSCs to form aggregations in differentiated medium, but not in stem cell media. These aggregates stained positively for toluidine blue and alcian blue, which stain cartilage extracellular matrices (Figures 11C, 11D) [35], [73]. For osteo-odontogenic differentiation, in contrast to undifferentiated cells, treated cells with differentiation media showed clusters of cells stained positively for DMP1 and DSP (Figures 11E–11G). The staining results were confirmed by RT-PCR (Figures 11H–11J). The gene expression after each differentiation revealed that induced cells expressed higher levels of specific adipogenic, chondrogenic, and osteo-odontogenic markers, respectively.

**Figure 12:**
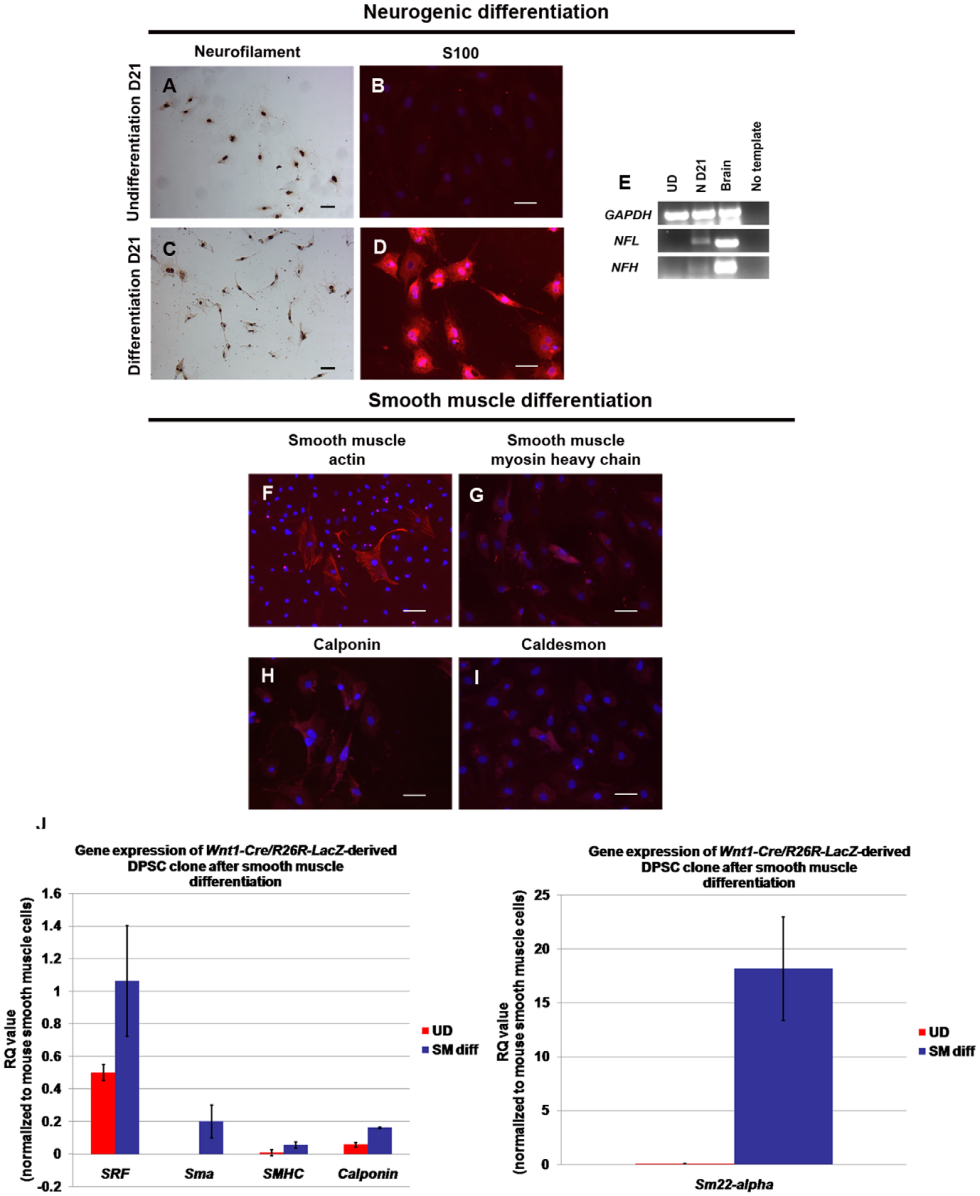
*Wnt1*-marked DPSCs gave rise into neural crest-derived non-mesenchymal lineages. (A–D) Neuronal-induced cells stained positively for neuronal markers; neurofilament and S100 (in red). (E) The neurofilament staining was confirmed by the expression of neurofilament-light (NFL) and -heavy (NFH) of treated cells in neurogenic media after 21 days (N D21) (F–I) DPSCs in smooth muscle differentiation media showed smooth muscle-like phenotype that stained positively in red fluorescence for smooth muscle actin, smooth muscle myosin heavy chain, calponin, caldesmon. DAPI used for nuclei staining is depicted in blue. (J) Corresponding to immunofluorescence, Q-RT-PCR revealed the up-regulation of smooth muscle-specific genes, *SRF, Sm22-alpha, Sma, SMHC,* and *calponin* in DPSCs after cultured in smooth muscle media. Scale bars indicate 100 µm. RQ values were normalized by the expression of mouse smooth muscle cells. *GAPDH* was used for the internal control. Error bars represent ±SEM.

In addition to mesenchymal lineages, clonal DPSCs gave rise into neuronal-like cells positive for neurofilament and S100, confirming by NFL and NFH gene expression (Figures 12A–12E). DPSCs cultured in smooth muscle media showed smooth muscle-like cells positive for smooth muscle actin; some of which stained positive for smooth muscle myosin heavy chain, calponin, and caldesmon, which are mature markers of smooth muscle cells (Figures 12F–12I) [53]. Q-RT-PCR showed treating DPSCs in smooth muscle media revealed up-regulation of specific gene expression for smooth muscle differentiation, *serum response factor (SRF), Sm22-alpha, Sma, SMHC*, and *calponin* (Figure 12J). Thus, *Wnt1*-marked DPSCs can successfully differentiate into neural crest lineages. Altogether these results confirm that clonal DPSCs generated in our culture conditions are all derived from neural crest origin.

**Figure 13:**
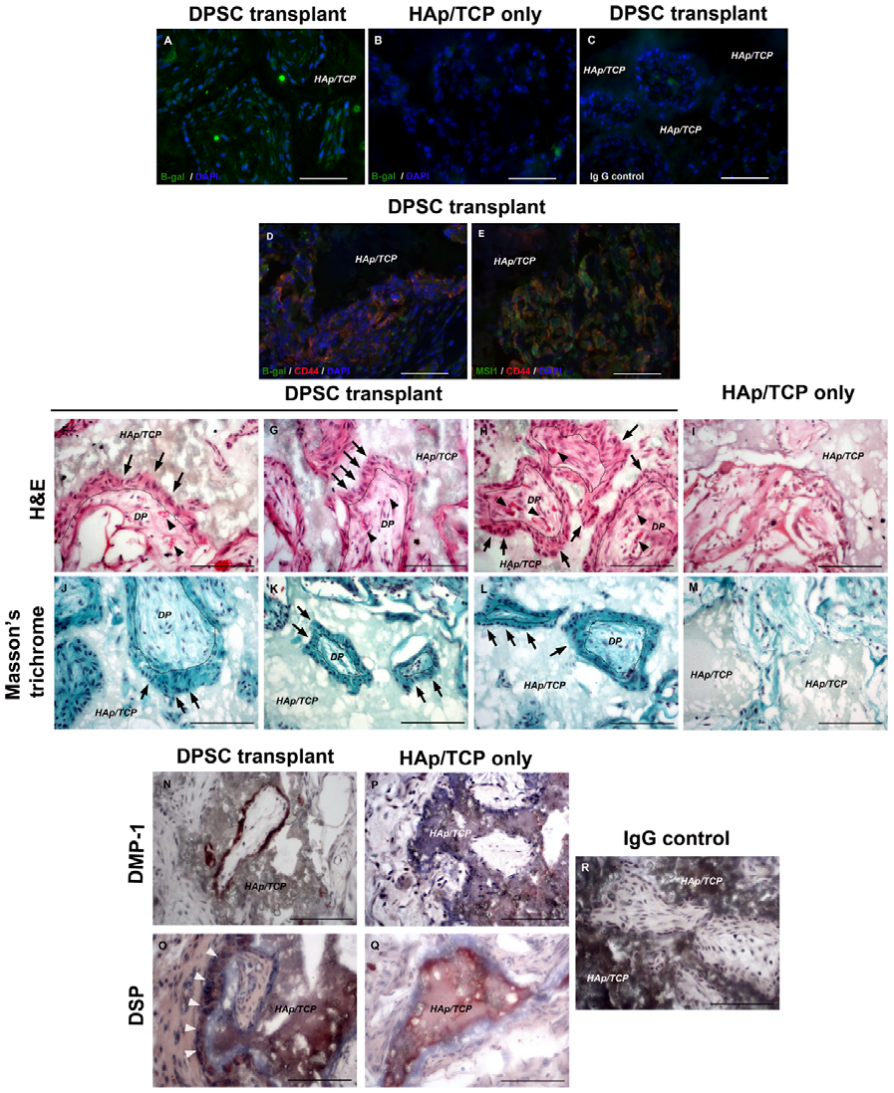
*Wnt1*-marked DPSCs gave rise to odontoblast-like cells and generated dentin-like structure in vivo. The *Wnt1*-marked DPSCs were subcutaneously transplanted in HAp/TCP scaffolds and analyzed 5 weeks post-transplantation. (A) anti β-galactosidase staining (in green) indicated the location of transplanted cells in DPSC transplantation. (B and C) Negative staining of anti β-galactosidase were observed in both transplantation of HAp/TCP only and IgG isotype control, respectively. (D and E) Co-localization of cells positive for β-galactosidase (in green) and CD44 (in red), as well as, MSI1 (in green) and CD44 (in red) were shown in odontoblast-like cells surrounding HAp/TCP scaffold surfaces in DPSC transplants. (F–H) H&E staining demonstrated the morphology of transplanted tissues in DPSC transplants. The transplanted DPSCs concentrated near HAp/TCP surfaces, and secreted extracellular matrices. Some of transplanted cells were elongated, polarized, and located perpendicularly to HAp/TCP surfaces, resembling odontoblast-like cells (indicated by arrows). Many loose connective tissue areas containing small blood vessels (indicated by arrowheads) surrounded by transplanted cells were found in DPSC transplants, resembling dental pulp-like tissues. (I) transplantations of HAp/TCP without cells were devoid of dense extracellular matrix. (J–M) Masson's trichrome staining confirmed the formation of collagen matrices found in DPSC transplants, but not in the transplantation of HAp/TCP only, respectively. (J–L) DPSC transplants showed elongated and polarized transplanted cells (indicated by arrows). (N–Q) DMP and DSP staining was strongly positive in DPSC transplants, but negative in the transplantation of HAp/TCP only, respectively. (O) White arrowheads illustrate polarized transplanted cells positive for DSP. (R) Staining with IgG isotype was used as negative control. DAPI used for nuclei staining is depicted in blue. The dot lines represent a boundary between extracellular matrices secreted by DPSCs and dental pulp-like structure. *DP*  =  Dental pulp-like structure, *HAp/TCP*  =  Hydroxyapatite/tricalcium phosphate. Scale bars indicate 100 µm.

Lastly, to determine the differentiation potential of cultured *Wnt1*-marked DPSCs *in vivo*, *Wnt1*-marked DPSCs were subcutaneously transplanted following the same protocol described above. Transplanted *Wnt1*-marked DPSCs were identified by anti-β-galactosidase staining in transplants (Figure 13A) whereas HAp/TCP only and transplanted tissue stained with IgG isotype control were negative for anti β-galactosidase staining (Figures 13B, 13C). Transplanted *Wnt1*-marked DPSCs were also positive for CD44 and MSI1, two markers expressed by DPSCs in dental pulp tissue and *in vitro* (Figures 13D, 13E).

*Wnt1-*marked DPSC concentrated near HAp/TCP areas, and secreted dense extracellular matrices. Some transplanted *Wnt1-*marked DPSCs were elongated, polarized, and arranged perpendicularly to HAp/TCP, resembling odontoblast-like cells (Figures 13F–13H, arrows). In addition, transplanted *Wnt1-*marked DPSCs also formed clusters of loose connective tissue containing blood vessels, resembling dental pulp-like tissue (Figures 13F–13H, arrowheads). As expected, transplanted HAp/TCP without DPSCs tissues were devoid of dense extracellular matrices (Figure 13I). Masson's trichrome confirmed dense collagen matrix formation in DPSC transplants, but not in HAp/TCP transplants only (Figures 13J–13M). In addition, DPSCs secreted extracellular matrices which were positive for dentin matrix proteins, DMP-1 and DSP (Figures 13N, 13O). The staining of dentin matrices were completely negative in the transplantation of HAp/TCP only (Figures 13P, 13Q). Corresponding to our previous results of DPSC transplantation, these observations confirmed that *Wnt1*-marked DPSCs generate odontoblast-like cells, dentin-like matrix and dental pulp-like tissue *in vivo*.

In addition to odontoblast-like cell differentiation, we found that some of DPSCs were strongly positive for smooth muscle actin, and those were located adjacent to blood vessels close to HAp/TC, suggesting pericyte-like phenotype (Figures S10A–S10F, arrowheads). DPSC-derived pericyte-like cells were also found surrounding blood vessels distant to HAp/TCP (Figures S10G–S10I, arrowheads). Nevertheless, some pericytes found in DPSC transplants were not derived from transplanted DPSCs as they did not stain positive for anti β-galactosidase. This transplantation model demonstrated that *Wnt1*-marked DPSCs were able to give rise not only odontoblast-like cells, but also pericyte-like cells.

## Discussion

Differences in DPSC proliferation and differentiation capacities *in vitro* and *in vivo* as described in previous reports indicate heterogeneity within DPSC populations [3], [6], [75]. In turn, such heterogeneity may be attributed to distinct developmental origins. The hypothesis that DPSCs are of neural crest origin has been suggested and indirectly related by previous reports [8]–[11]. Accordingly, human dental pulp contains self-renewing DPSCs capable of giving rise to mesenchymal-lineages and non-mesenchymal cells such as neuron-like, smooth muscle-like, and melanocyte-like cells, indicating that these cells might be derived from neural crest [3], [76], [77]. Herein we explored several criteria to determine if stem cells in dental pulp originate from neural crest. First we investigated the proliferative capacity of murine DPSCs cultured in stem cell selective media, as well as, the expression of stem cell and neural crest genes. Next, we determined *in vitro* and *in vivo* multi-differentiation of DPSCs into neural crest-lineage. Lastly, we used the *Wnt1-Cre/R26R-LacZ* transgenic mouse to conclusively determine that DPSCs generated in our cultures are of neural crest origin.

Unlike human, mouse DPSCs have not been well described possibly due to the difficult nature of isolating dental pulp from murine teeth [2]. A recent study characterized progenitors and stem cells in dental pulps of mouse molars in uneruption and eruption states *in vitro* and revealed a small population of mesenchymal multipotent cells in erupted molars but not in the dental pulp of the unerupted molars. Instead, a majority of dental pulp cells of the unerupted molar were osteo-dentogenic progenitor cells [78]. In our studies, we determined the existence of stem cells in mouse dental pulp from unerupted molars which exhibit high proliferation and multi-differentiation into mesenchymal and non-mesenchymal lineages. DPSCs sufficiently self-perpetuated more than 14 passages (∼3 months) without morphological changes. DPSCs chosen for all differentiation experiments were harvested near passage 7, the time which *Nanog* and *Klf4* expression was highest (Figure S5). In this study we used the optimal conditions for long-term cultivation of stem cells without any signs of degeneration or spontaneous differentiation; near physiological oxygen concentration, and a low concentration of serum supplemented with platelet derived growth factor-BB (PDGF-BB), epidermal growth factor (EGF), insulin transferrin selenium (ITS), dexamethasone, and leukemia inhibitory factor (LIF) [15], [79]. This combination differs from previously described culture condition used to grow human and mouse DPSCs [2], [78]. Therefore, a direct comparison of culture conditions must be undertaken to determine if DPSC described here are equivalent to previously described populations.

Interestingly the expression of embryonic pluripotent genes suggests the existence of primitive stem cells within the DPSC population that share a similar genetic program to embryonic and inducible pluripotent stem cells [16]. Q-RT-PCR results showed down-regulation of *Klf4* and *Nanog* after differentiation of DPSCs, suggesting that both transcription factors play a similar role and function for maintenance of DPSC multi-potentiality and undifferentiated state. Consistent with our hypothesis of a neural crest origin, DPSCs expressed multiple neural crest markers whereas they did not show mesodermal developmental markers. To determine that DPSCs fulfill the stem cell criteria, in addition to self-renewal, we also explored their multi-differentiation capacity with emphasis on neural crest lineages.

Explants of cranial and trunk neural crest cells give rise to chondrocytes, glia, neuronal cells, smooth muscle cells, and melanin-forming cells, which are all neural crest-lineage derived during embryogenesis [13]. In our differentiation conditions, treated DPSCs expressed markers for osteoblast, chondrocyte, neuron, smooth muscle and adipocyte differentiation, indicating that non-clonal DPSCs contains multipotent stem cells with capacity to differentiate into mesenchymal and non-mesenchymal lineages of neural crest cells. Consistently human DPSCs can *in vitro* differentiate into non-mesenchymal lineages [3], [76].

DPSC clones, initiated from early passages of non-clonal cultures, were sub-cultured for 1 month without changing cell morphology. Like non-clonal DPSCs, all clones expressed *Klf4*, *Nanog*, and neural crest-related genes, suggesting that clones maintain neural-crest multipotential. Our observations showed DPSC clones gave rise to odontoblast-like, chondrocyte-like, adipocyte-like, smooth muscle-like, and neuronal-like cells.

To confirm the neural crest origin of DPSCs, we used a genetic lineage tracing model of neural crest derivatives. Although the *Wnt1-Cre/R26R-LacZ* mouse has been widely used to study neural crest development, to date the origin of DPSCs has not been examined [9]. Staining results of *Wnt1-Cre/R26R-LacZ* tooth sections showed the existence of neural crest-derived DPSCs in sub-odontoblastic and perivascular regions. Those two areas have been reported as stem cell niches in dental pulp tissue [70]–[72]. Under our culture conditions, early dental pulp cells isolated from the *Wnt1-Cre/R26R-LacZ* revealed the majority of cells were of neural crest origin. This is consistent with the study by Chai et al (2000) showing that dental pulp consists of cells from neural crest and non-neural crest origins [9]. In addition, *Wnt1-Cre/R26R-LacZ*-derived BMSCs, previously known as non-neural crest-derived used as a control showed negative X-gal staining [62].

The X-gal staining of both DPSCs and BMSCs indicates that the *Wnt1-Cre/R26R-LacZ* is a good strategy to study the developmental origin of DPSCs. This model definitely confirms the neural crest origin of DPSCs. In spite of a mixed cell population of DPSC in early culture, after 6-week-*in vitro* expansion, all cells were positive by X-gal staining indicating only neural crest-derived cells remained and were present in late cultures. The *Wnt1-Cre/R26R-LacZ*-derived DPSC clones also expressed strongly pluripotent stem cell and neural crest-related genes, and gave rise to neural crest lineages. In addition to the expression of neural crest genes and differentiation capacity into neural crest-lineage, this lineage tracing data undeniably proves that in our culture condition DPSCs are neural crest-derived stem cells.

To further assess DPSC plasticity, we transplanted cultured DPSCs to examine their response *in vivo* in two different models: subcutaneous (SC) and intramuscular (IM) transplantations. Interestingly, each transplantation method resulted in different cell fates indicating that DPSCs are not under a fixed program as environmental factors influence their response. Short subcutaneous transplantation with HAp/TCP resulted in the production of a disorganized dentin-like structure, mimicking a structure generated in reparative dentin formation after tooth injury [69]. Reparative dentin exhibits disorganized arrangement due to lack of signaling from inner enamel epithelium and basement membrane which results in multiple inducing molecules secreted from dentin matrix [71]. Lack of epithelium induction may explain why DPSC clones generated a disorganized structure. Like the renal capsule or subcutaneous regions, we used the intramuscular model to transplant DPSCs since we hypothesized that a high vascularized system in muscle may enhance the function of DPSCs [2], [3], [80]. In turn, IM transplantation of DPSCs generated well-organized but immature collagen-forming matrix. These results may be explained by several possibilities. First, each transplant environment has a distinct influence on differentiation of DPSCs. Secondly, the IM transplanted structure was more organized possibly because muscle fibers function as scaffold to stabilize and directly instruct transplanted cells toward the proper arrangement and orientation. Thirdly, the length of transplantation in the IM injections was only 2 weeks, 3 weeks shorter than SC transplantation, and thus, longer IM transplantation may result in more mature matrix formation.

To determine if longer transplantations are required for formation of more mature matrices, we performed IM and SC transplantations for 12 weeks. Surprisingly, longer *in vivo* exposure did not result in more mature matrix formation in the intramuscular transplants. However, longer subcutaneous transplantations with HAp/TCP resulted in more mature matrix formation in both DPSC and BMSC transplants but the morphology and matrix compositions differed.

First, we observed many abundant small blood vessels in close proximity to odontoblast-like cells in DPSC transplants. Many of these microvessels were fenestrated and stained positive for VEGFR-3. We observed pericyte-like cells adjacent to these microvessels; some of which were donor DPSC-derived. This observation illustrates the perivascular nature of DPSCs and their potential to function as pericytes [81]. Although BMSC transplants also contained abundant microvessels, bone condensations were significantly distant to vessels. Interestingly, in BMSC transplantations we observed a large number of microvessels in areas rich in adipocytes. These cells may be from BMSC-derived or derived from host cells which were signaled by BMSCs to differentiate into adipocytes.

Secondly, long-term DPSC transplantation resulted in a more organized mineralized matrix formed by elongated and polarized cells resembling odontoblast-like morphology which were in close proximity to microvessels. These matrices represent dentin-like structure with strong positive staining for dentin matrix proteins, DMP1, DSP, and BSP. In contrast, BMSC transplantation showed mineralized matrices with osteocyte-like cells in lacunae. Consistently, the matrices produced by BMSCs were positive for the bone protein, BSP, but not positive for the dentin protein, DSP. However, some studies reported that certain states of bone or tooth development can express low levels of dentin or bone proteins, respectively; therefore, we cannot distinguish bone or dentin structures by only protein expression [31], [49]. As mentioned above, the combination of cell/tissue morphology and matrix protein expression is important to characterize the structure created by DPSCs and BMSCs [2], [50], [80].

Thirdly, as described previously in human DPSC *in vivo* transplantation, murine DPSCs can create collagen fibers arranging perpendicularly to the scaffold/matrix surface whereas BMSCs created collagen fibers running parallel to the scaffold/matrix surface [2], [3]. Lastly, the distribution and association of odontoblasts with microvessels resembles dentinogenesis whereas the formation of bone condensation in avascular areas resembles early osteogenesis [57]–[59].

**Figure 14:**
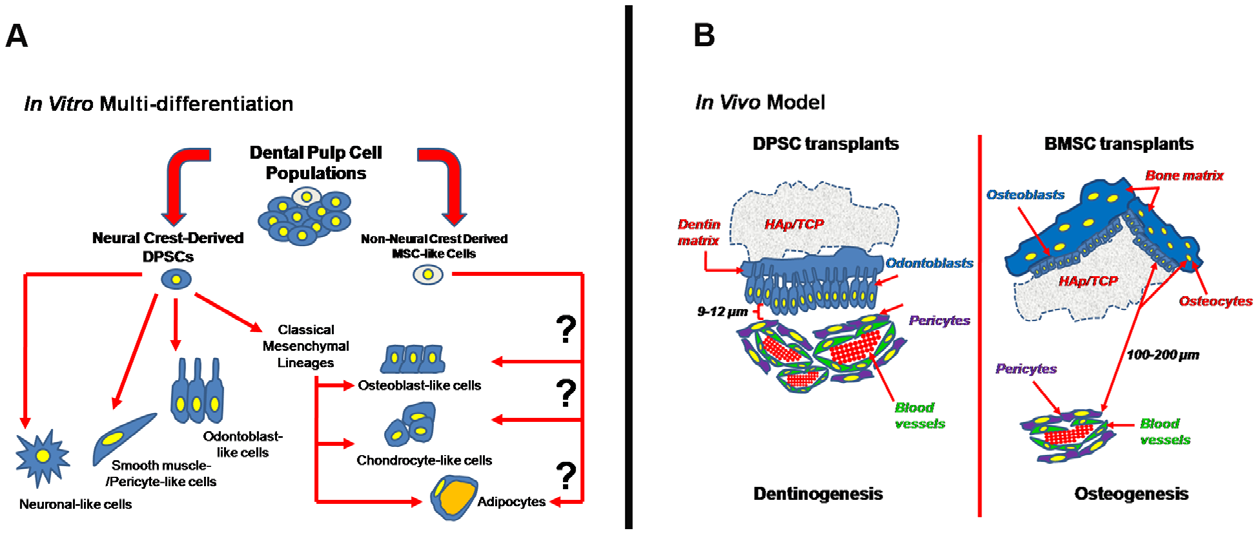
Summary of results and working models of neural crest-derived stem cells isolated from murine dental pulp tissue. (A) Base on the *Wnt1-Cre/R26R-LacZ* mouse model, neural crest gene expression, and differentiation capacity, dental pulp stem cell (DPSC) populations contain a majority of neural crest-derived cells (approximately 90%) (shown in blue) while the remaining cell populations were derived from non-neural crest origin (shown in white). In our condition, only neural crest-derived cells can survive and proliferate. After *in vitro* differentiation, these cells can give rise to neural crest-lineages, neuronal-like, smooth muscle/pericyte-like, odontoblast-like cells, including classical mesenchymal cell lineages, osteoblast-like, chondrocyte-like, and adipocytes. Nonetheless, in this study the differentiation capacity of non-neural crest-derived mesenchymal stem cell (MSC)-like cells was not characterized. (B) *In vivo* models show the different capacity between DPSCs and bone marrow stromal cells (BMSCs). Based on the cell/tissue morphology and antibody staining, DPSCs formed dentin-like matrix composed of odontoblast-like cells and pericytes associated with microvessels (close distance about 9–12 µm) recapitulating dentinogenesis. On the other hand, the formation of bone condensation from BMSCs occurred in avascular areas (far distant, about 100–200 µm), resembling early osteogenesis. This model illustrates how two different types of mesenchymal stem cells derived from different origins form different niches and matrices when transplanted under the same conditions. Interestingly, these tissue-specific stem cells recapitulate formation of their own tissue (organogenesis). Understanding the mechanisms of this “memory” of their tissues of origin may be pivotal information for tissue regeneration and cell therapy.

In summary, we showed dental pulp cells in mouse neonates with neural crest-related stem cell properties. Although some studies showed that both neural crest and non-neural crest cells can be found in dental pulp during tooth development, our stem cell conditions select for only the outgrowth of neural crest-derived stem cells (Figure 14A). Thus, future studies should aim at comparing the differentiation capacity, contribution and cross-talk of neural crest- and non-neural crest-derived cells from dental pulp. Upon transplantation, DPSCs formed dentin-like matrix composed of odontoblast-like cells and pericytes associated with microvessels in dental pulp-like tissue recapitulating dentinogenesis (Figure 14B).

The mechanisms that control the differentiation fate of dental pulp stem cells are not completely elucidated but interactions with their niche are important for maintenance of stemness and induction/guidance for differentiation. In our transplantation model, interactions with microvessels seem pivotal for the differentiation of DPSCs into pericytes and odontoblasts. Since the dental pulp tissue is highly vascularized, it is probable that dental pulp stem cells reside in a vascular niche. To date multiple reports have revealed the presence of various adult stem cells residing within the vascular niches [70], [82]–[84].

In addition, several recent studies have shown the existence of neural crest stem cells in various postnatal tissues [27], [85], [86]. Thus, future studies should also aim at understanding the interactions of neural crest-derived stem cells with their respective niches from multiple adult tissues. Understanding the mechanisms that control organogenesis and adult tissue homeostasis will be instrumental for tissue engineering and tissue regeneration. Thus, studying how neural crest-derived stem cells from multiple organs interact within each niche to remain either stem cells or differentiate may help elucidate the mechanisms for maintenance of tissue-specific stem cells and tissue- specific differentiation. This will broaden the potential clinical applications of DPSCs as a readily accessible source for tissue regeneration not only of pulp regeneration but possibly other neural crest derivatives.

## Materials and Methods

### Ethics Statement

All mouse experiments and protocols were performed in accordance with approved Institutional Animal Care and Use Committee (IACUC) guidelines.

### Dental pulp isolation

Dental pulp tissue were isolated in pools (5 mice per preparation) from 4–10 day-old neonatal *Tie2*-GFP (Jackson Laboratory) (n = 3 different preparations) and *Wnt1-Cre/R26R-LacZ* mice (n = 1 preparation) (a gift from Dr. Michael Cunningham, University of Washington) in accordance with approved Institutional Animal Care and Use Committee (IACUC) guidelines. The molar teeth were separated from the mandible. Pulp tissue was gently isolated and kept in stem cell media described below. The tissue was washed with phosphate buffer saline (PBS) (HyClone) and digested with a 1.2 units/ml dispase II, 2 mg/ml collagenase type IV (Worthington) supplemented with 2 mM CaCl_2_ in PBS for 60 min at 37°C. Subsequently an equal volume of stem cell media was added to the digest prior to filtering through 70 mm nylon cell strainers (BD Falcon), and then centrifuging at 300 g for 10 min at room temperature. Cells were then resuspended in stem cell media and single cell suspensions were plated at 1000 cells/cm^2^.

### Culture of DPSCs

Cells (1000 cells/cm^2^) were cultured at 37°C under 5% O_2_ and 5% CO_2_ in stem cell media, containing a final concentration of 60% low-glucose DMEM (Gibco, Invitrogen), 40% MCDB201 (Sigma), 2% fetal calf serum (HyClone) selected previously for optimal growth of murine mesenchymal stem cells, insulin-transferrin-selenium(ITS) ( (Sigma), linoleic acid with bovine serum albumin (LA-BSA) (Sigma), 10^−9^ M dexamethasone (Sigma), 10^−4^ M ascorbic acid 2-phosphate (Sigma), 100 units/ml penicillin with 100 mg/ml streptomycin (HyClone), and 1×10^3^ units/ml leukemia-inhibitory factor (LIF-ESGRO, Millipore), supplemented with 10 ng/mL EGF (Sigma) and 10 ng/mL PDGF-BB (R&D) [79]. Once adherent cells were more than 50% confluent, they were detached with 0.25% trypsin-EDTA (Invitrogen) and replated at a 1∶4 dilution under the same culture condition with fresh media.

### Clonal culture of DPSCs

Cultured DPSCs at passage 4–7 were plated at 50-100 cells/cm^2^. Clones were derived from all three non-clonal lines but only clones from line 1 at passage 7 were used in the experiments described here. 24 h after plating, adherent cells were observed and single isolated cells were marked by circling the bottom of the plate with a lab marker. After 10 days, cell colonies were observed and isolated by using 8×8 mm cloning rings (Millipore). The clonal populations were expanded every 4 days and transferred to larger size culture dishes beginning with 96-well, 48-well, 24-well, 12-well, 6-well, 60 mm and 100 mm respectively, until finally being seeding in 150 mm culture plates by day 32. The DPSC clones were cultured at a 1∶4 dilution until reaching the appropriate cell number for further experiments. The *Wnt1-Cre/R26R-LacZ* derived DPSCs were cloned and expanded following the protocol described above.

### RT-PCR and Q-RT-PCR analyses

DPSCs were extracted for total RNA by using the RNeasy Mini kit (Qiagen) according to the manufacturer's protocol. Quantity and purity of RNA was determined by 260/280 nm absorbance. First-strand cDNA was synthesized from 500 ng of RNA using the High Capacity cDNA synthesis kit from Applied Biosystems per manufacturer's protocols using a randomized primer. RT-PCR and Q-RT-PCR protocol details and primers are included in Text S1 and Table S2, respectively.

### In vitro multi-differentiation

The compositions of each differentiation media and cell densities are listed in Text S1 and Table S3. DPSCs were plated at corresponding cell density in 24-well plates (BD) and incubated overnight in stem cell media at 37°C under 5% O_2_ and 5% CO_2_. After 24 h, media was switched to corresponding differentiation media for 21 days with media change every 3 days. In addition to monolayer differentiation, for chondrogenic differentiation *Wnt1-Cre/R26R-LacZ* derived DPSCs were cultured in chondrogenic media by 3D-pellet culture technique. The 3D-pellet culture is one of widely used technique for chondrogenic differentiation [73]. Briefly, 2×10^5^ cells were transferred in a 15-ml polypropylene conical tube and centrifuged at 450 g for 10 min. The pellets were cultured at 37°C under 5% O_2_ condition in 1 ml of chondrogenic media for 21 days with media change every 3 days. After 21 days, the pellets were fixed 4% formaldehyde/PBS for 30 min and wash with PBS. Then, fixed pellets were embedded in optimal cutting temperature compound (OCT) (Tissue-Tek; Sakura Finetek), frozen with liquid nitrogen cooled isobutane and cut to 8–10 µm thick sections.

### Staining of DPSCs

For immunocytochemistry, cells were fixed with ice cold methanol for 5 min, permeabilized with 1% BSA in 0.1% Triton-X 100 (Sigma)/PBS for 10 min, inhibited endogenous peroxidase activity with 0.3% hydrogen peroxide in methanol for 30 min, and blocked non-specific binding sites with 10% goat or horse serum (Vector Burlingame, CA) for 1 h. All primary antibodies listed in Table S4 were used and incubated overnight at 4°C. Stained cells were incubated with a biotinylated antibody at 1∶100 (Vector Burlingame, CA) for 1 h, washed and treated with the Vectastain ABC kit and 3, 3′-diaminobenzidine (DAB) or 3-amino-9-ethylcarbazole (AEC) substrate kit according to manufacturers protocol (Vector Burlingame, CA). For immunofluorescence, cells were fixed with 4% formaldehyde/PBS for 5 min, washed with 1% BSA in 0.1% Triton-X 100/PBS, and stained with primary antibodies as described above. Donkey-derived Alexa 488 or goat-derived Alexa 594-conjugated secondary antibodies (Invitrogen) were diluted at 1∶800 and incubated for 1 h. Cells were stained with 4′, 6-diamine-2-phenylindol (DAPI) at 1∶1000 to visualize the nuclei. All antibodies were diluted in 1% BSA in 0.1% Triton-X 100/PBS. We used two types of controls, omitting the primary antibody and IgG isotype from the species made for the primary antibody (0.1 µg/ml) (Vector Burlingame, CA), were included for all staining. Toluidine blue and alcian blue were performed to determine proteoglycan producing cells in chondrogenic culture [87]. Oil Red O was used for characterizing lipid-containing cells in adipogenic culture [87]. X-gal was stained for determining the expression of *LacZ* gene in *Wnt1*-marked DPSCs [87].

### Tooth histology and staining

Mandibles were isolated from 4–10 day-old *Tie2-GFP* or *Wnt1-Cre/R26R-LacZ* mice and cut in halves after removing surrounding connective tissues. To preserve GFP, *Tie2-GFP* derived mandibles were fixed with 0.5% formaldehyde/PBS for 2 h at RT, and washed. The first wash was 30 min followed by 20-min and 10-min washes, respectively [88]. For *Wnt1-Cre/R26R-LacZ* derived mandibles, they were fixed with 4% formaldehyde/PBS for 2 h at 4°C, and wash. After washing, mandibles from both transgenic mice were immersed in 20% sucrose in PBS at 4°C overnight to preserve tissue morphology before embedding in OCT and frozen with liquid nitrogen cooled isobutane. Since those mandibles were incompletely calcified, we did not process decalcification. Therefore, we cut the tissues into 15–20 µm thickness to get a good morphology of tissue section.

For immunofluorescence of tooth section, we did double staining between β-galactosidase or MSI1 and CD44. Briefly, tooth sections were fixed with 4% formaldehyde/PBS for 10 min at RT. Fixed sections were washed and permeabilized with 1% BSA in 0.1% Triton X-100/PBS. Then sections were blocked with 10% normal goat serum for 1 h at RT , and incubated with rabbit-anti-mouse-β-galactosidase or -MSI1 for 1 h at RT following three times of washing. Stained sections were subsequently incubated with goat-derived Alexa 488 or goat-derived Alexa 647-conjugated secondary antibody (Invitrogen) at 1∶800 dilution for 1 h at RT, following three times of washing. Then sections were incubated by the second set of primary antibody which is rat-anti-mouse-CD44 for 1 h at RT, washed, and incubated with goat-derived Alexa 594-conjugated secondary antibody (Invitrogen) at 1∶800 dilution for 1 h at RT before washing three times. All immunofluorescence images described in this manuscript was detected using a Zeiss Axiovert 200 fluorescent microscope (Thornwood, NY). Photographs were taken with an onboard monochrome AxioCam MRm camera and colored using Adobe Photoshop (San Jose, CA). Background was reduced using brightness and contrast adjustments, and color balance was performed to enhance colors. All the modifications were applied to the whole image using Adobe Photoshop.

### In vivo transplantation

In accordance with approved IACUC protocols, 1×10^6^ of murine non-clonal DPSCs, clonal DPSCs, and BMSCs were separately transplanted into 1-month-old male *Rag1* null mice (Jackson Laboratory, Bar Harbor, ME, USA) by two methods (n = 3 mice/cell line); 1) intramuscular (IM) injection in the tibialis anterior, and 2) subcutaneous (SC) transplantation with hydroxyapatite tricalciumphosphate (HAp/TCP) (Zimmer) in the dorsum. BMSCs were isolated and cultured as previously described [89]. Prior to transplantation, cells were labeled with the fluorescent membrane dye PKH-26 (Sigma) according to manufacturer's instructions. Grafts were harvested after 2-wk and 12-wk IM, 5-wk and 12-wk SC transplantations. For SC-transplanted tissues, samples were fixed with 4% formaldehyde for 2 h then demineralized for 7 days in 10% EDTA at 4°C. Both IM- and SC-transplanted tissues were embedded in OCT, frozen with liquid nitrogen cooled isopentane and cut to 10–13 µm thick sections. Some SC-transplants were embedded in paraffin and cut to 5 µm thick sections. Sections were analyzed by H&E, Masson's trichrome, immunohistochemistry, and immunofluorescence [87].

## Supporting Information

Figure S1 **DPSC isolation and culture.** (A and B) First and second developing molar teeth of 4–10 day-old neonatal mice were isolated from mandible. (C) DPSCs started to proliferate and form a small colony after 2 days in stem cell media under 5% O_2_ incubation. (D) DPSCs at day 10 formed larger colonies and were confluent. (E) The cell morphology of DPSCs in early cultures was heterogenous; most of which were spindle-shaped cells. T  =  teeth, and M  =  mandible. Scale bars indicate 200 µm.
(TIF)

Figure S2 **Gene and protein expression of undifferentiated DPSCs.** Immunocytochemistry showed positive staining of KLF4 to confirm the pluripotency gene *Klf4* expression, and of neural crest-related proteins, MSI1 and SOX10. KLF4 and SOX10 showed nuclear and perinuclear staining. Perinuclear localization of KLF4 and SOX10 has been described in previous reports [7], [8]. Staining with IgG isotype was used as negative control. RT-PCR also showed the expression of *Sox10* in undifferentiated cells. RNA of salivary gland was used as positive control while the reaction without cDNA was used as negative control. Scale bars indicate 100 µm.
(TIF)

Figure S3 **Down-regulation of** ***Nanog*** **and** ***Klf4*** **after differentiation of DPSCs.** Q-RT-PCR analyses showed *Nanog* and *Klf4* were down-regulated after osteogenic and chondrogenic differentiation of DPSC line 1. RQ (Relative Quantification) values were normalized by the expression of mouse embryonic stem cells (mESCs). Similar results were observed for DPSC line 2. *GAPDH* was used for the internal control. Error bars represent ±SEM.
(TIF)

Figure S4 **DPSC clonal isolation and gene profile.** (A–D) At day 10, colonies derived from single cells can be visualized. (E and F) Gene expression of cells from fresh dental pulp (Tissue), cultured non-clonal DPSC line 1 (Non-clonal), and different DPSC clones (C5–C9). (E) RT-PCR showed variable *Nanog* and stable *Klf4* expression among different clones. All clones showed the absence of early mesodermal genes *Brachyury* and *Mesp2*, but showed expression of neural crest developmental genes *Pax3, Gsc,* and *GATA6*. (F) Several neural crest-related genes *Msi1, Twist, Slug, Snail,* and *Pdgfra*, and mesenchymal-related gene *Vimentin*, were strongly expressed in clones. *Dmp1*, but not *Dspp*, was also observed in the clones. RNA of mouse embryonic stem cells (mESCs), salivary gland, clavarial bone and cementoblast cell line were used as positive control while the reaction without cDNA was used as negative control. Scale bars indicate 300 µm.
(TIF)

Figure S5 ***Nanog*** **and** ***Klf4*** **expression of undifferentiated non-clonal and clonal DPSCs.** Q-RT-PCR showed RQ (Relative Quantification) values demonstrating differential *Nanog* and *Klf4* expression in fresh dental pulp (Tissue), non-clonal (bulk) DPSCs in several passages (P0, P3, and P7), as well as, DPSC clones derived from the non-clonal populations (C5–C9). The non-clonal DPSC passage 7 showed the highest expression of *Nanog* and *Klf4* among different passages of cultured non-clonal DPSCs, which we chose for differentiation assays. Nevertheless, dental pulp tissue expressed higher level of *Klf4* than that of cultured cells, possibly due to contamination of odontoblasts expressing *Klf4* during pulp isolation but thereafter the stem cell-like population probably outgrew primary mature odontoblasts under stem cell culture conditions [7]. DPSC clones 6, 7, and 8 showed higher expression of *Nanog* as compared to non-clonal populations. These clones showed neural crest multi-lineage differentiation capacity (Table S1). C5 and C9 showed higher levels of *Nanog* and *Klf4* than that of C7 and C8 but were not able to differentiate into most neural crest-lineages (Table S1). Consequently, DPSC clones 6, 7 and 8 were chosen for *in vivo* transplantation. RQ values were normalized by the expression of mouse embryonic stem cells (mESCs). *GAPDH* was used for the internal control. Error bars represent ±SEM.
(TIF)

Figure S6 **Gene expression of non-clonal and clonal DPSCs after smooth muscle differentiation.** Q-RT-PCR showed RQ (Relative Quantification) values demonstrating smooth muscle genes of non-clonal and clonal DPSCs after 21-day culture in smooth muscle differentiation media. As compared to non-clonal DPSCs, higher expression of *Sm22-alpha*, *Sma*, *SMHC*, and *Calponin,* which are smooth muscle- and pericyte-related genes, were observed in the undifferentiated clonal DPSCs. The differentiated cells derived from the clonal populations showed a pattern of smooth muscle maturation with significantly increased levels of *Myocardin, Sm22a, Sma*, *SMHC*, and *Calponin* (about 10 folds higher than non-clonal differentiated cells and >100 folds compared to aorta smooth muscle cells) while in the non-clonal populations this trend of maturation is not apparent. RQ values were normalized by the expression of mouse smooth muscle cells (a gift from Dr. William Mahoney Jr., University of Washington). *GAPDH* was used for the internal control. Error bars represent ±SEM.
(TIF)

Figure S7 **Intramuscular transplantation of DPSCs and BMSCs.** (A) Non-clonal DPSCs were identified as PKH-26 positive cells. (B, D–F) 2- or 12-week intramuscular non-clonal DPSC, clonal DPSC, and BMSC transplants showed donor cells formed compacted collagen bundles which were strongly positive for Masson's trichrome in blue, but slight positive for DMP1, DSP, and OCN (data not shown). Skeletal muscles were shown in red. (C, G-I) Polarized light confirmed the formation of collagen fibers from all transplanted cells. Scale bars indicate 100 µm.
(TIF)

Figure S8 **Staining of tooth sections for dentin and bone proteins.** The specificity of all antibodies for dentin and bone matrix proteins used in this report was confirmed by immunoperoxidase staining with AEC in murine tooth sections as positive control. All tooth sections are a gift from Dr. Martha Somermen, University of Washington. (A and B) The tooth section of 19-day-old Ankylosis (ANK) knockout mice stained by DMP1 antibody showed positive staining in cementum and alveolar bone [9]. (C-L) Staining of the 26-day-old wild-type (WT) murine tooth section showed positive staining of DSP, BSP, and OCN, as well as, negative controls. (C and D) DSP staining was present in dentin and odontoblast cells lining at the periphery of dental pulp. (E–H) BSP staining was positive in alveolar bone and cementum, but slightly positive in odontoblastic cell layer, as well as, OCN positively stained alveolar bone and odontoblastic cell layer. (I–L) Sections stained with anti-rabbit biotinylated antibody or IgG isotype were used as negative control. Sections were counterstained with hematoxylin. AB  =  Alveolar bone, DT  =  Dentin, DP  =  Dental pulp, C  =  Cementum, PDL  =  Periodontal ligament. Scale bars indicate 200 µm.
(TIF)

Figure S9 **Abundant microvessels were observed in DPSC transplantation.** (A, inset, indicated by an arrowhead) Abundant microvessels that stained positive for CD31-FITC indicating endothelial cells (green) were observed in mineralized tissues formed by 5-week subcutaneous transplanted DPSCs labeled with PKH26 (red). Some PKH-26+ (donor DPSCs) cells were adjacent to these microvessels and seem scattered around vessels. (B) Autoflurescence or non-specific green fluorescence was not detected in PKH26-labeled DPSC transplants without CD31-FITC staining. (C–F, inset) Staining for smooth muscle actin (SMA-FITC) in green showed that these microvessels did not contain a thick muscle layer but were wrapped by pericyte-like cells that were SMA+ and some were also PKH-26+ in red (arrowheads). Many of these microvessels seem fenestrated and stained positive for VEGF receptor 3 in pseudo-color white. (G, inset) Similar to 5-week transplants, in the 12-week DPSC transplants abundant microvessels (positive for VEGF receptor 3 in pseudo-color magenta) in close proximity to odontoblast-like cells with fenestrated morphology surrounded by PKH-26+ cells (indicated by arrows) were observed in DPSC transplants. (H, inset) In the BMSC transplants, microvessels were predominantly found in areas rich in adipocytes, but not close to the bone forming areas. DAPI used for nuclei staining is depicted in blue. Scale bars indicate 100 µm.
(TIF)

Figure S10 ***Wnt1*** **-marked DPSCs gave rise to pericyte-like cells** ***in vivo*** **.** (A–I) Double staining of anti β-galactosidase (B-gal, in green) and smooth muscle actin (SMA, in red) showed that some transplanted DPSCs labeled by anti B-gal were positive for SMA (indicated by white arrowheads) and located adjacent to blood vessels (indicated by red arrows), indicating pericyte-like phenotype. Some SMA-negative cells were elongated and polarized lining the HAp/TCP surface, representing odontoblast-like cells (indicated by white arrows). (A, D, and G, as well as B, E, and H) Monochromatic figures represented anti β-galactosidase and smooth muscle actin staining, respectively. (C, F, and I) Fluorescence staining was merged to illustrate pericyte-like cells that co-stained positive for anti β-galactosidase and smooth muscle actin (white arrowheads) although some non-specific green and red fluorescence was detected in red blood cells and HAp/TCP, respectively. DAPI used for nuclei staining is depicted in blue. *BV*  =  Blood vessel, *HAp/TCP*  =  Hydroxyapatite/tricalcium phosphate. Scale bars indicate 100 µm.
(TIF)

Table S1 **Variable differentiation capacity of non-clonal and clonal DPSCs.** Table S1 shows the summary and comparison of the capacity of non-clonal DPSCs and clones to differentiate into neural crest-derived mesenchymal (osteogenic, odontogenic, adipogenic, and chondrogenic) and non-mesenchymal (smooth muscle and neuronal) lineages. These data indicate that non-clonal DPSCs were able to give rise to all neural crest-lineages. In contrast, DPSC clones differentiated at passage 7 showed differentiation capacity into certain lineages, but lack osteogenic and adipogenic capacity. Nonetheless, the clones still showed multi-differentiation capacity into mesenchymal and non-mesenchymal lineages. Interestingly, DPSC clones isolated from the *Wnt1-Cre/R26R-LacZ* differentiated at passage 4 show the capacity to differentiate into osteoblasts and adipocytes, which differs from that of our previous clones. This may indicate DPSCs lose differentiation capacity to certain lineages during long-term cultures. In addition, differences in the differentiation capacity among the clones indicate heterogeneity or hierarchical relationship of stem cells and progenitors. Note that POS (positive) means that cells can give rise to that lineage whereas NEG (negative) means that cells did not differentiate into that lineage.
(DOC)

Table S2 **The mouse-specific primer sequences.**
(DOC)

Table S3 **Lists of differentiation media.**
(DOC)

Table S4 **Lists of antibody for staining.**
(DOC)

Text S1 **Supporting Information Methods and Supporting Information Reference.**
(DOC)
