# Supplementary figures and images for: Isolation and Characterization of Neural Crest-Derived Stem Cells from Dental Pulp of Neonatal Mice
Source: PLoS One. 2011 Nov 8;6(11):e27526. doi: 10.1371/journal.pone.0027526 (PMC3210810; doi:10.1371/journal.pone.0027526)

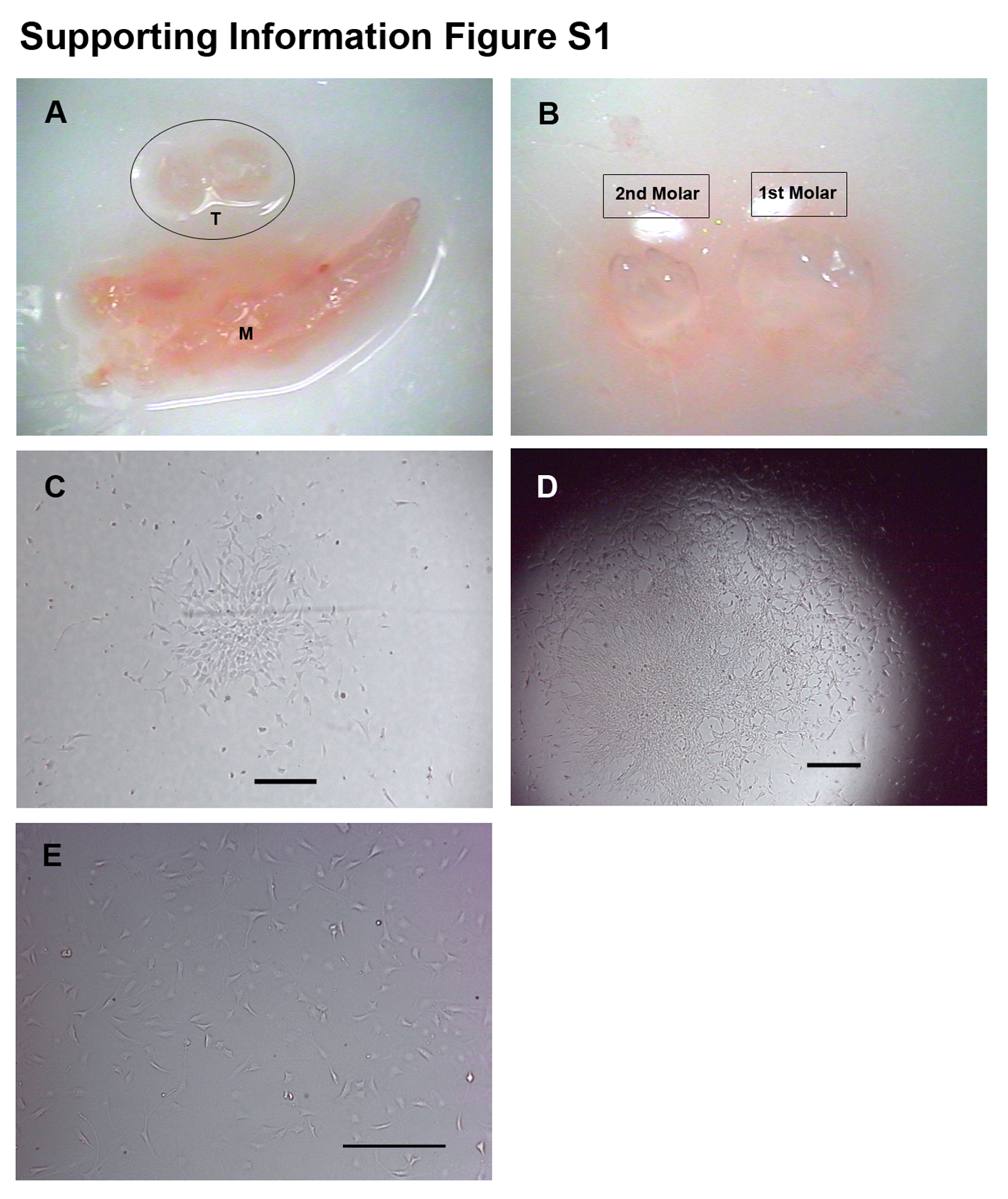

Supplement: Figure S1 — DPSC isolation and culture. (A and B) First and second developing molar teeth of 4–10 day-old neonatal mice were isolated from mandible. (C) DPSCs started to proliferate and form a small colony after 2 days in stem cell media under 5% O2 incubation. (D) DPSCs at day 10 formed larger colonies and were confluent. (E) The cell morphology of DPSCs in early cultures was heterogenous; most of which were spindle-shaped cells. T = teeth, and M = mandible. Scale bars indicate 200 µm. (TIF) [file pone.0027526.s001.tif]

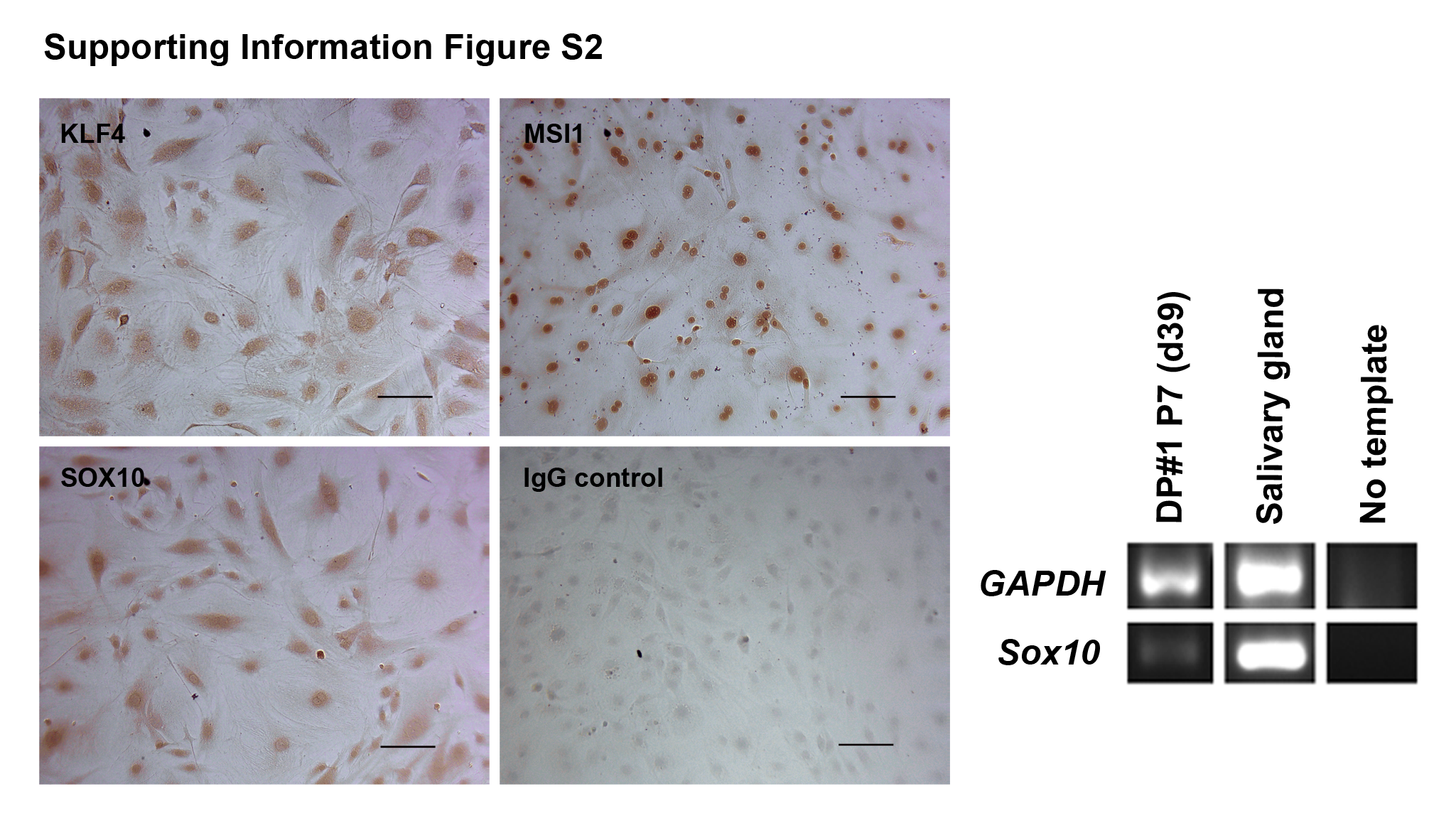

Supplement: Figure S2 — Gene and protein expression of undifferentiated DPSCs. Immunocytochemistry showed positive staining of KLF4 to confirm the pluripotency gene Klf4 expression, and of neural crest-related proteins, MSI1 and SOX10. KLF4 and SOX10 showed nuclear and perinuclear staining. Perinuclear localization of KLF4 and SOX10 has been described in previous reports [7], [8]. Staining with IgG isotype was used as negative control. RT-PCR also showed the expression of Sox10 in undifferentiated cells. RNA of salivary gland was used as positive control while the reaction without cDNA was used as negative control. Scale bars indicate 100 µm. (TIF) [file pone.0027526.s002.tif]

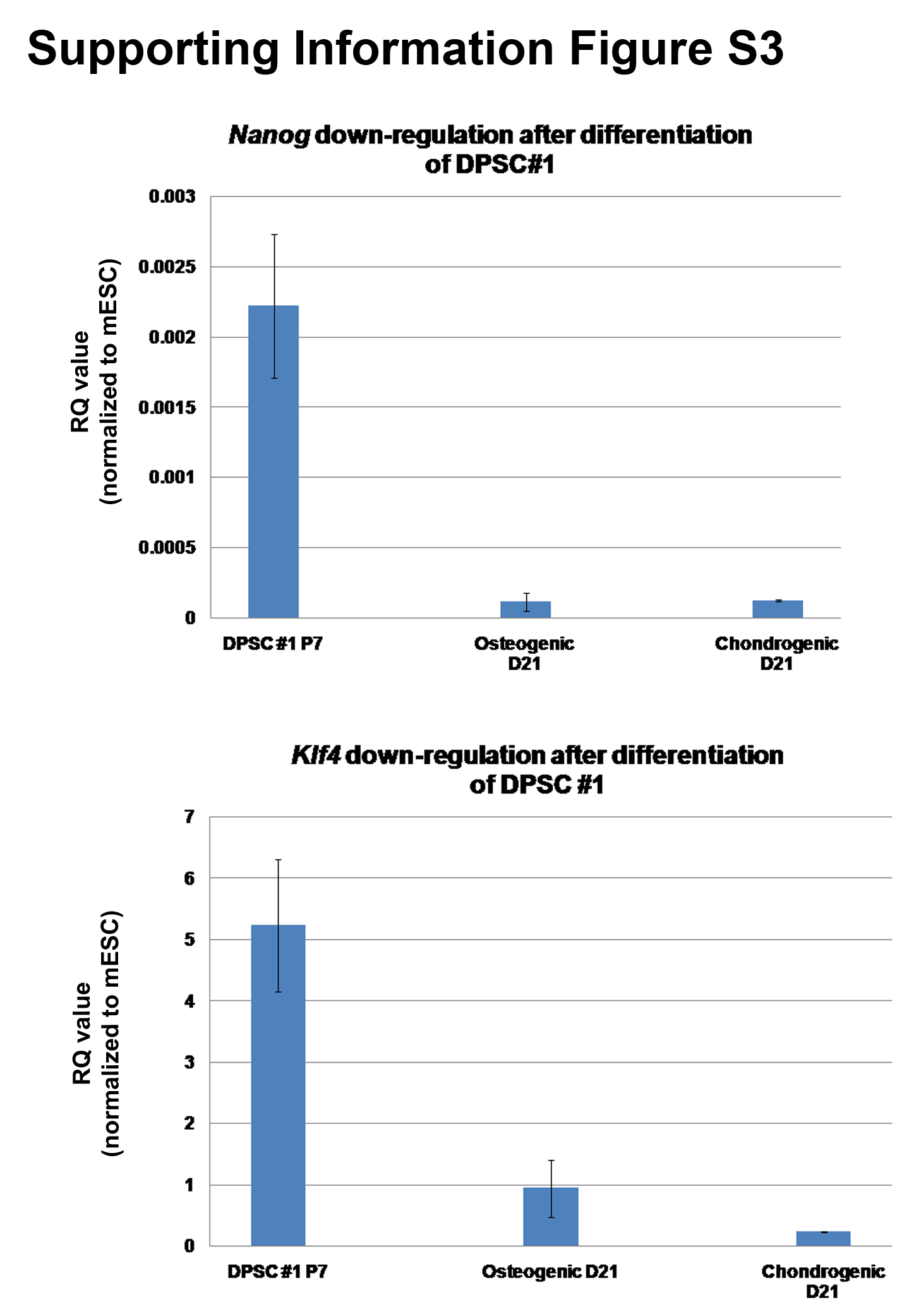

Supplement: Figure S3 — Down-regulation of Nanog and Klf4 after differentiation of DPSCs. Q-RT-PCR analyses showed Nanog and Klf4 were down-regulated after osteogenic and chondrogenic differentiation of DPSC line 1. RQ (Relative Quantification) values were normalized by the expression of mouse embryonic stem cells (mESCs). Similar results were observed for DPSC line 2. GAPDH was used for the internal control. Error bars represent ±SEM. (TIF) [file pone.0027526.s003.tif]

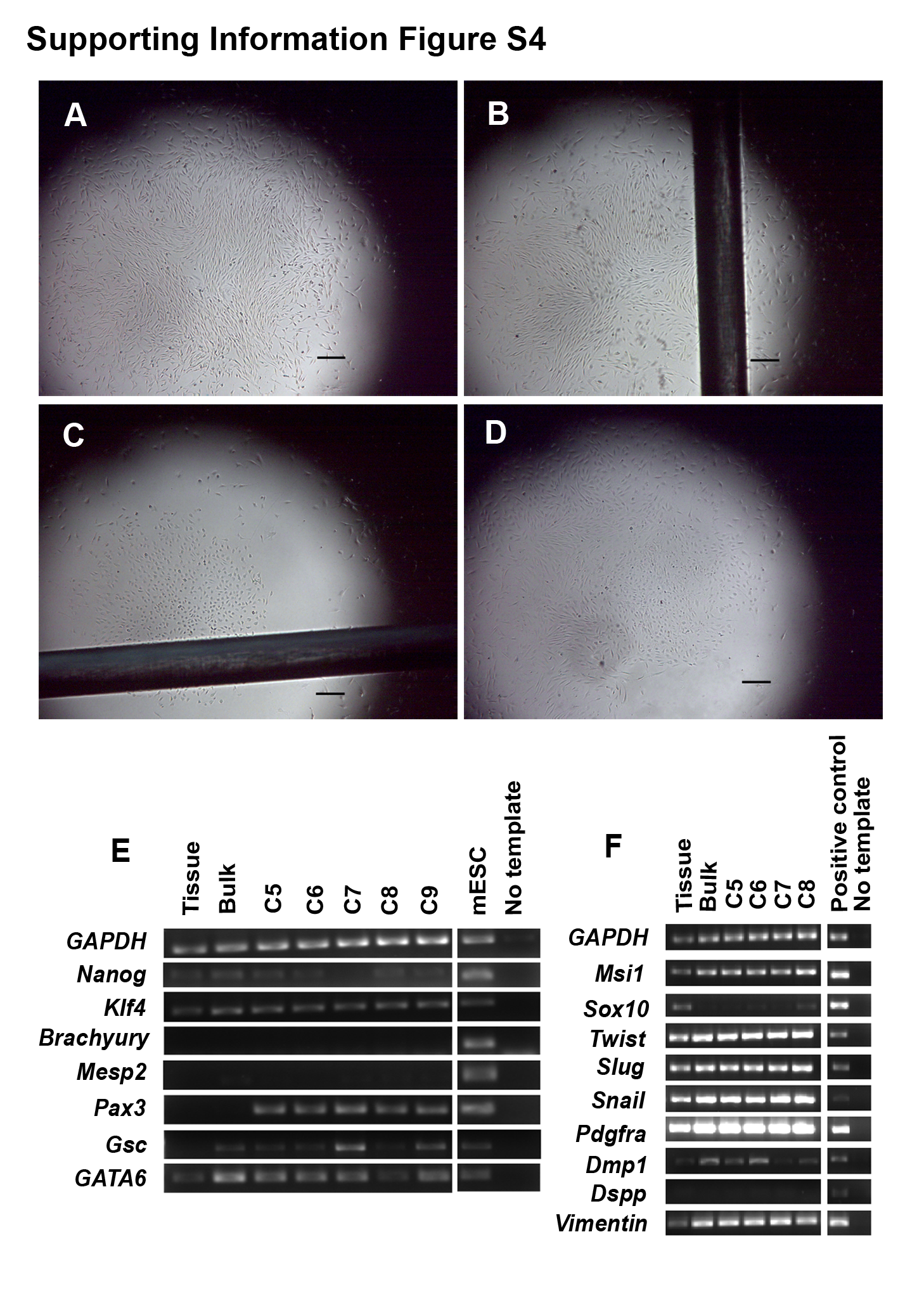

Supplement: Figure S4 — DPSC clonal isolation and gene profile. (A–D) At day 10, colonies derived from single cells can be visualized. (E and F) Gene expression of cells from fresh dental pulp (Tissue), cultured non-clonal DPSC line 1 (Non-clonal), and different DPSC clones (C5–C9). (E) RT-PCR showed variable Nanog and stable Klf4 expression among different clones. All clones showed the absence of early mesodermal genes Brachyury and Mesp2, but showed expression of neural crest developmental genes Pax3, Gsc, and GATA6. (F) Several neural crest-related genes Msi1, Twist, Slug, Snail, and Pdgfra, and mesenchymal-related gene Vimentin, were strongly expressed in clones. Dmp1, but not Dspp, was also observed in the clones. RNA of mouse embryonic stem cells (mESCs), salivary gland, clavarial bone and cementoblast cell line were used as positive control while the reaction without cDNA was used as negative control. Scale bars indicate 300 µm. (TIF) [file pone.0027526.s004.tif]

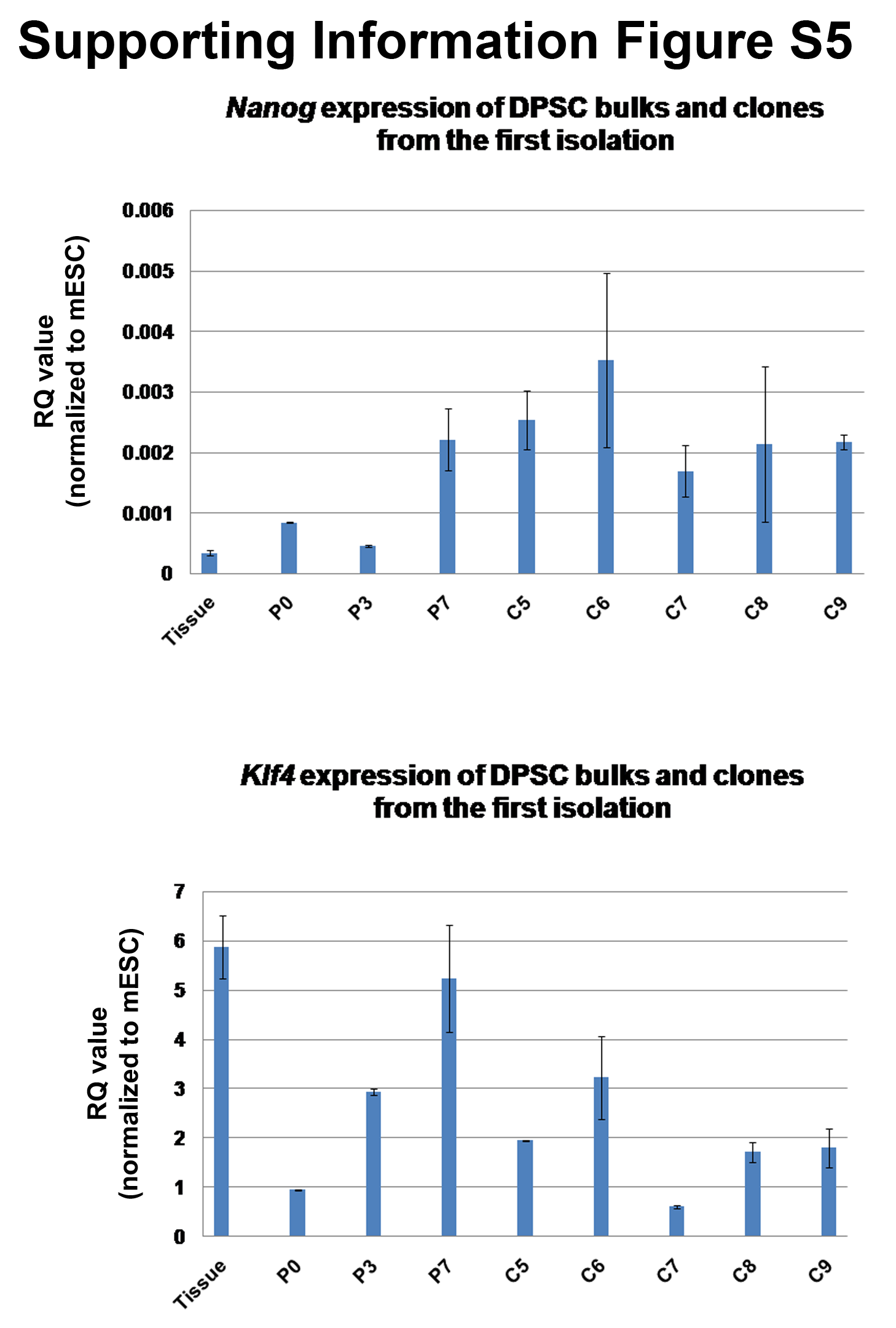

Supplement: Figure S5 — Nanog and Klf4 expression of undifferentiated non-clonal and clonal DPSCs. Q-RT-PCR showed RQ (Relative Quantification) values demonstrating differential Nanog and Klf4 expression in fresh dental pulp (Tissue), non-clonal (bulk) DPSCs in several passages (P0, P3, and P7), as well as, DPSC clones derived from the non-clonal populations (C5–C9). The non-clonal DPSC passage 7 showed the highest expression of Nanog and Klf4 among different passages of cultured non-clonal DPSCs, which we chose for differentiation assays. Nevertheless, dental pulp tissue expressed higher level of Klf4 than that of cultured cells, possibly due to contamination of odontoblasts expressing Klf4 during pulp isolation but thereafter the stem cell-like population probably outgrew primary mature odontoblasts under stem cell culture conditions [7]. DPSC clones 6, 7, and 8 showed higher expression of Nanog as compared to non-clonal populations. These clones showed neural crest multi-lineage differentiation capacity (Table S1). C5 and C9 showed higher levels of Nanog and Klf4 than that of C7 and C8 but were not able to differentiate into most neural crest-lineages (Table S1). Consequently, DPSC clones 6, 7 and 8 were chosen for in vivo transplantation. RQ values were normalized by the expression of mouse embryonic stem cells (mESCs). GAPDH was used for the internal control. Error bars represent ±SEM. (TIF) [file pone.0027526.s005.tif]

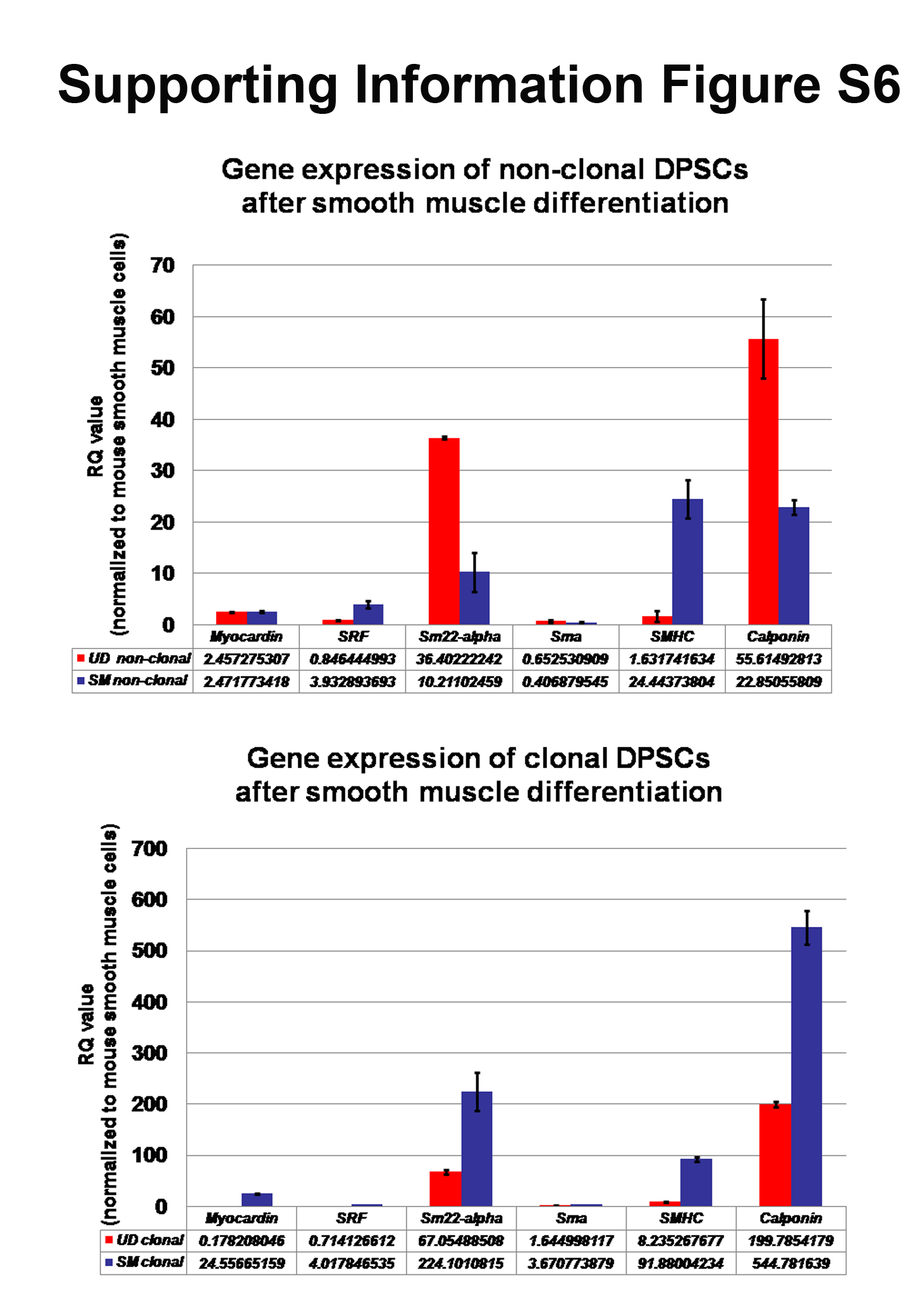

Supplement: Figure S6 — Gene expression of non-clonal and clonal DPSCs after smooth muscle differentiation. Q-RT-PCR showed RQ (Relative Quantification) values demonstrating smooth muscle genes of non-clonal and clonal DPSCs after 21-day culture in smooth muscle differentiation media. As compared to non-clonal DPSCs, higher expression of Sm22-alpha, Sma, SMHC, and Calponin, which are smooth muscle- and pericyte-related genes, were observed in the undifferentiated clonal DPSCs. The differentiated cells derived from the clonal populations showed a pattern of smooth muscle maturation with significantly increased levels of Myocardin, Sm22a, Sma, SMHC, and Calponin (about 10 folds higher than non-clonal differentiated cells and >100 folds compared to aorta smooth muscle cells) while in the non-clonal populations this trend of maturation is not apparent. RQ values were normalized by the expression of mouse smooth muscle cells (a gift from Dr. William Mahoney Jr., University of Washington). GAPDH was used for the internal control. Error bars represent ±SEM. (TIF) [file pone.0027526.s006.tif]

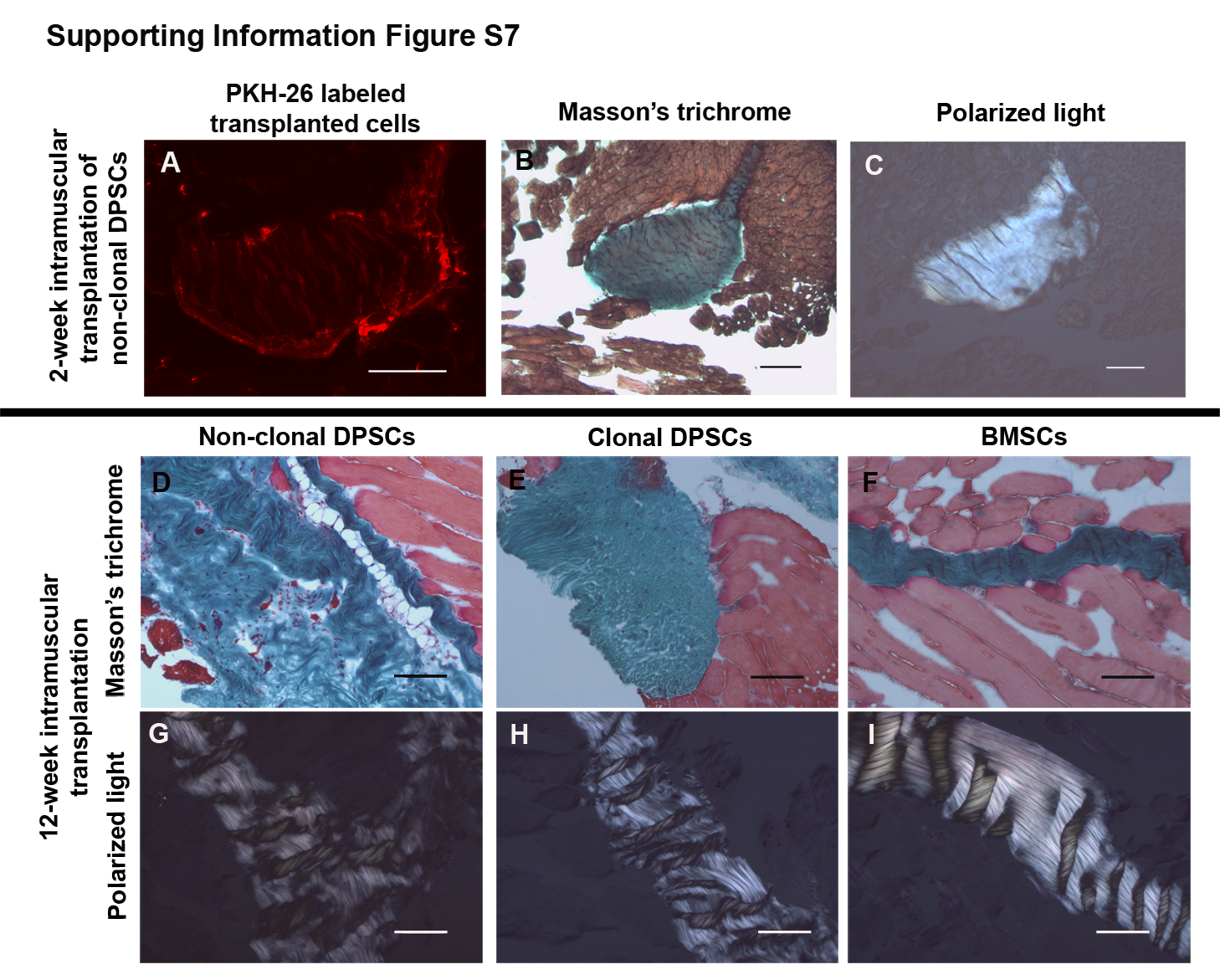

Supplement: Figure S7 — Intramuscular transplantation of DPSCs and BMSCs. (A) Non-clonal DPSCs were identified as PKH-26 positive cells. (B, D–F) 2- or 12-week intramuscular non-clonal DPSC, clonal DPSC, and BMSC transplants showed donor cells formed compacted collagen bundles which were strongly positive for Masson's trichrome in blue, but slight positive for DMP1, DSP, and OCN (data not shown). Skeletal muscles were shown in red. (C, G-I) Polarized light confirmed the formation of collagen fibers from all transplanted cells. Scale bars indicate 100 µm. (TIF) [file pone.0027526.s007.tif]

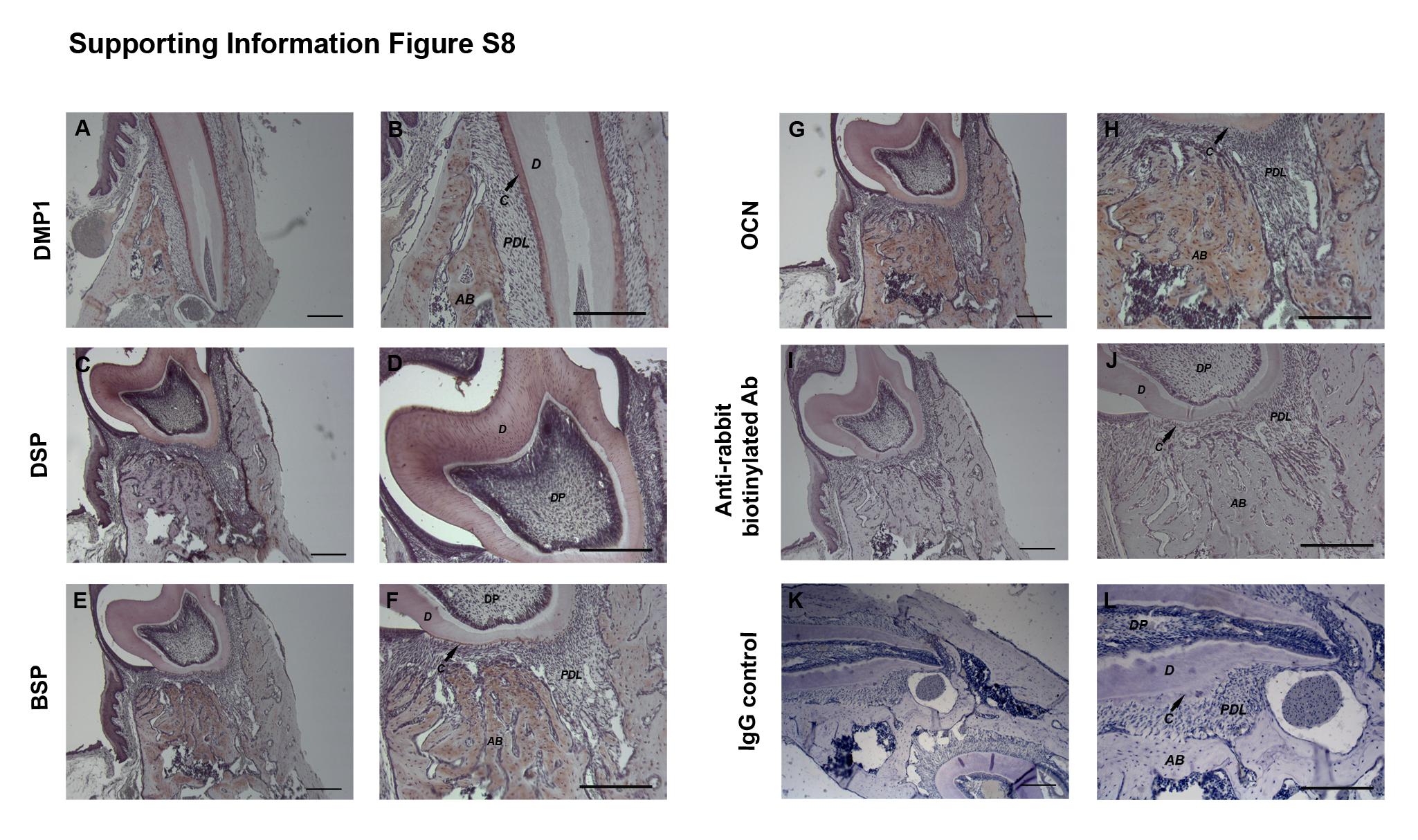

Supplement: Figure S8 — Staining of tooth sections for dentin and bone proteins. The specificity of all antibodies for dentin and bone matrix proteins used in this report was confirmed by immunoperoxidase staining with AEC in murine tooth sections as positive control. All tooth sections are a gift from Dr. Martha Somermen, University of Washington. (A and B) The tooth section of 19-day-old Ankylosis (ANK) knockout mice stained by DMP1 antibody showed positive staining in cementum and alveolar bone [9]. (C-L) Staining of the 26-day-old wild-type (WT) murine tooth section showed positive staining of DSP, BSP, and OCN, as well as, negative controls. (C and D) DSP staining was present in dentin and odontoblast cells lining at the periphery of dental pulp. (E–H) BSP staining was positive in alveolar bone and cementum, but slightly positive in odontoblastic cell layer, as well as, OCN positively stained alveolar bone and odontoblastic cell layer. (I–L) Sections stained with anti-rabbit biotinylated antibody or IgG isotype were used as negative control. Sections were counterstained with hematoxylin. AB = Alveolar bone, DT = Dentin, DP = Dental pulp, C = Cementum, PDL = Periodontal ligament. Scale bars indicate 200 µm. (TIF) [file pone.0027526.s008.tif]

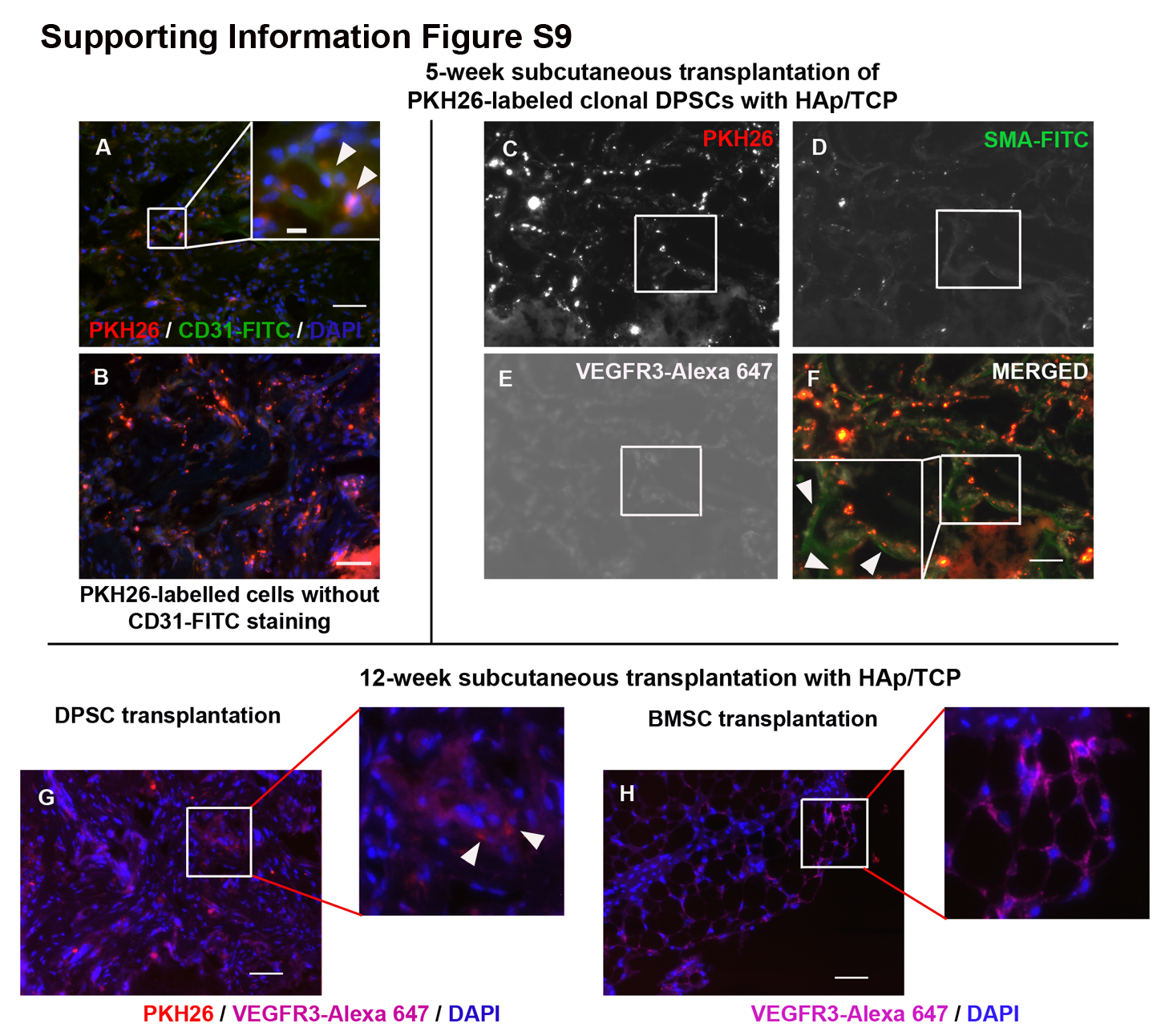

Supplement: Figure S9 — Abundant microvessels were observed in DPSC transplantation. (A, inset, indicated by an arrowhead) Abundant microvessels that stained positive for CD31-FITC indicating endothelial cells (green) were observed in mineralized tissues formed by 5-week subcutaneous transplanted DPSCs labeled with PKH26 (red). Some PKH-26+ (donor DPSCs) cells were adjacent to these microvessels and seem scattered around vessels. (B) Autoflurescence or non-specific green fluorescence was not detected in PKH26-labeled DPSC transplants without CD31-FITC staining. (C–F, inset) Staining for smooth muscle actin (SMA-FITC) in green showed that these microvessels did not contain a thick muscle layer but were wrapped by pericyte-like cells that were SMA+ and some were also PKH-26+ in red (arrowheads). Many of these microvessels seem fenestrated and stained positive for VEGF receptor 3 in pseudo-color white. (G, inset) Similar to 5-week transplants, in the 12-week DPSC transplants abundant microvessels (positive for VEGF receptor 3 in pseudo-color magenta) in close proximity to odontoblast-like cells with fenestrated morphology surrounded by PKH-26+ cells (indicated by arrows) were observed in DPSC transplants. (H, inset) In the BMSC transplants, microvessels were predominantly found in areas rich in adipocytes, but not close to the bone forming areas. DAPI used for nuclei staining is depicted in blue. Scale bars indicate 100 µm. (TIF) [file pone.0027526.s009.tif]

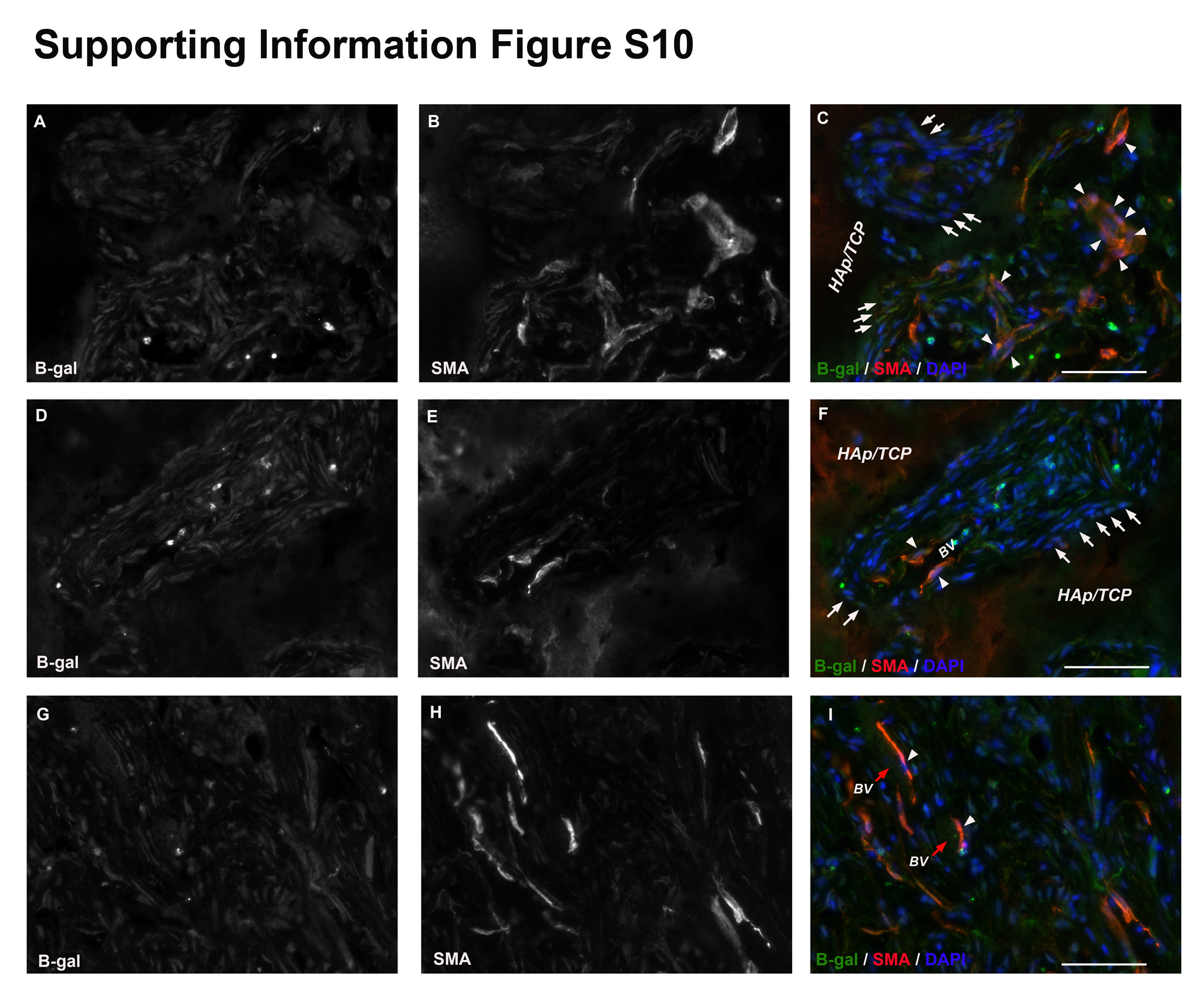

Supplement: Figure S10 — Wnt1 -marked DPSCs gave rise to pericyte-like cells in vivo . (A–I) Double staining of anti β-galactosidase (B-gal, in green) and smooth muscle actin (SMA, in red) showed that some transplanted DPSCs labeled by anti B-gal were positive for SMA (indicated by white arrowheads) and located adjacent to blood vessels (indicated by red arrows), indicating pericyte-like phenotype. Some SMA-negative cells were elongated and polarized lining the HAp/TCP surface, representing odontoblast-like cells (indicated by white arrows). (A, D, and G, as well as B, E, and H) Monochromatic figures represented anti β-galactosidase and smooth muscle actin staining, respectively. (C, F, and I) Fluorescence staining was merged to illustrate pericyte-like cells that co-stained positive for anti β-galactosidase and smooth muscle actin (white arrowheads) although some non-specific green and red fluorescence was detected in red blood cells and HAp/TCP, respectively. DAPI used for nuclei staining is depicted in blue. BV = Blood vessel, HAp/TCP = Hydroxyapatite/tricalcium phosphate. Scale bars indicate 100 µm. (TIF) [file pone.0027526.s010.tif]
