# Supplementary material for: Isolation and Characterization of Neural Crest-Derived Stem Cells from Dental Pulp of Neonatal Mice
Source: PLoS One. 2011 Nov 8;6(11):e27526. doi: 10.1371/journal.pone.0027526 (PMC3210810; doi:10.1371/journal.pone.0027526)
Supplement: Table S1 — Variable differentiation capacity of non-clonal and clonal DPSCs. Table S1 shows the summary and comparison of the capacity of non-clonal DPSCs and clones to differentiate into neural crest-derived mesenchymal (osteogenic, odontogenic, adipogenic, and chondrogenic) and non-mesenchymal (smooth muscle and neuronal) lineages. These data indicate that non-clonal DPSCs were able to give rise to all neural crest-lineages. In contrast, DPSC clones differentiated at passage 7 showed differentiation capacity into certain lineages, but lack osteogenic and adipogenic capacity. Nonetheless, the clones still showed multi-differentiation capacity into mesenchymal and non-mesenchymal lineages. Interestingly, DPSC clones isolated from the Wnt1-Cre/R26R-LacZ differentiated at passage 4 show the capacity to differentiate into osteoblasts and adipocytes, which differs from that of our previous clones. This may indicate DPSCs lose differentiation capacity to certain lineages during long-term cultures. In addition, differences in the differentiation capacity among the clones indicate heterogeneity or hierarchical relationship of stem cells and progenitors. Note that POS (positive) means that cells can give rise to that lineage whereas NEG (negative) means that cells did not differentiate into that lineage. (DOC) [file pone.0027526.s011.doc]

**Table S1. Variable differentiation capacity of non-clonal and clonal DPSCs.**

| **Differentiation / Clone** | **Non-clonal**  **#1** | **Non-clonal**  **#2** | **C5** | **C6** | **C7** | **C8** | **C9** | ***Wnt1*-marked DPSC clone** |
| --- | --- | --- | --- | --- | --- | --- | --- | --- |
| Osteogenic | POS | POS | NEG | NEG | NEG | NEG | NEG | POS |
| Odontogenic | POS | POS | POS | POS | POS | POS | POS | POS |
| Adipogenic | POS | POS | NEG | NEG | NEG | NEG | NEG | POS |
| Chondrogenic | POS | POS | POS | POS | POS | POS | NEG | POS |
| Smooth muscle | POS | POS | POS | POS | POS | POS | POS | POS |
| Neurogenic | POS | POS | NEG | POS | POS | POS | NEG | POS |
