# Supplementary material for: Isolation and Characterization of Neural Crest-Derived Stem Cells from Dental Pulp of Neonatal Mice
Source: PLoS One. 2011 Nov 8;6(11):e27526. doi: 10.1371/journal.pone.0027526 (PMC3210810; doi:10.1371/journal.pone.0027526)
Supplement: Table S2 — The mouse-specific primer sequences. (DOC) [file pone.0027526.s012.doc]

**Table S2. The mouse-specific primer sequences**

| **RT-PCR primers** | |  |  |  |
| --- | --- | --- | --- | --- |
| **Gene** | **Forward primer**  **(5’🡪3’)** | | **Reverse primer**  **(5’🡪3’)** | **GenBank Accession number** |
| *Adipsin* | GTGCTGCACACTGCATGGAT | | CCGGGTTCCACTTCTTTGTC | NM_013459 |
| *Brachyury* | CCCCTATGCTCATCGGAACA | | GTAGGTGGGCTGGCGTTATG | NM_009309 |
| *Bsp* | AGAACAATCCGTGCCACTCACT | | CCCTGGACTGGAAACCGTTT | NM_008318 |
| *CD105* | CTTCGTACAGGTGAGCGTGTCT | | GCTAGGGCGCAGAGCTAAGTT | NM_007932 |
| *CEBPa* | GCCAAGAAGTCGGTGGACAA | | AGTTGCCCATGGCCTTGAC | NM_007678 |
| *c-Kit* | AGCCTGGCGTTTCCTACG T | | GCCCGAAATCGCAAATCT TT | NM_001122733 |
| *c-Myc* | CCTCTGCCCGCGATCA | | AGGAAATCCAGCCTTCAAACAG | NM_010849 |
| *Col2a1* | AGGATGGCTGCACGAAACA | | TCAGTGCAGATCCTGGAGTGACT | NM_031163 |
| *Dmp1* | TTGGGAGCCAGAGAGGGTAGA | | AGTCCACCAGCCGGTCTGTA | NM_016779 |
| *Dspp* | CCCCTCGGAGGCTTTGA | | ACTCGGAGCCATTCCCATCT | NM_010080 |
| *GAPDH* | CTCGTCCCGTAGACAAAATGG | | CGCTCCTGGAAGATGGTG | NM_008084 |
| *GATA6* | AGCAGGACCCTTCGAAACG | | GCGCTTCTGTGGCTTGATG | NM_010258 |
| *Gsc* | ACGCTGTCGCGCACTGA | | CTCCGGCGAGGCTTTTG | NM_010351 |
| *Klf4* | GGTTTTGGTTTGAGGTTTTGTTTCT | | CCTCACGCCAACGGTTAG TC | NM_010637 |
| *Leptin* | ACCTGCTCCGGGTACATGTTC | | TGGGCAGACCCATCAATAGG | NM_008493 |
| *LNGFR* | GAGGCACCGCTGACAACCT | | CAGGCCTCGTGGGTAAAGG | NM_033217 |
| *Lpl* | GGATGGACGGTAACGGGAAT | | CATGGGCTCCAAGGCTGTAC | NM_008509 |
| *Mesp2* | CCCCAAATACAGTCACCCTTACAC | | GGCTGTAGTCTCTGGCATGATG | NM_008589 |
| *Msi1* | GACCCCTGCAAGATGTTCATC | | CTCTGTGCCTGTTGGTGGTTT | NM_008629 |
| *Nanog* | TCTCAAGTCCTGAGGCTGACAAG | | GTGCTGAGCCCTTCTGAATCA | NM_028016 |
| *NCAM* | TGCTCGTGTGTCCTCCTTGA | | GCTTGGCAGCAACTGACCAT | NM_001081445 |
| *NFH* | CGTAAAACACGCGTCTAAAAACTG | | GAGTACACCCTGGCGTGG TT | NM_010904 |
| *NFL* | GCCTTGGACATCGAGATTGC | | CAGCTTTCGTAGCCTCAATGG | NM_010910 |
| *Ocn* | TTGGTGCACACCTAGCAGACA | | TCGTCACAAGCAGGGTTAAGC | NM_001032298 |
| *Oct4* | CTGGGCGTTCTCTTTGGAAA | | TCGGGCACTTCAGAAACATG | NM_013633 |
| *Opn* | CAGTGATTTGCTTTTGCCTGTT | | TCGTCGTCCATGTGGTCATG | NM_009263 |

| **RT-PCR primers** | | |  | |  |  |
| --- | --- | --- | --- | --- | --- | --- |
| **Gene** | **Forward primer**  **(5’🡪3’)** | | | | **Reverse primer**  **(5’🡪3’)** | **GenBank Accession number** |
| *Osx* | AGAGATCTGAGCTGGGTAGAGGAA | | | | AAGTTGAGGAGGTCGGAGCAT | NM_130458 |
| *Pax3* | CGCTGTCTGTGATCGGAACA | | | | TCTGCTCCTGCGCTGCTT | NM_001159520 |
| *Pdgfra* | TTTGTGCCTCTCGGGATGA | | | | TGACGGGCAGCACATTCA | NM_011058 |
| *Pparg2* | CAAGAATACCAAAGTGCGATCAAA | | | | GGATCCGGCAGTTAAGATCACA | NM_011146 |
| *Runx2* | AATGCCTCCGCTGTTATGAAA | | | | GAATGCGCCCTAAATCACTGA | NM_001146038 |
| *Slug* | CACTGTGATGCCCAGTCTAGGA | | | | GCAGATGTGCCCTCAGGTTT | NM_011415 |
| *Snail* | TGACCTCGCTGTCCGATGA | | | | GTGCTTGTGGAGCAAGGACAT | NM_011427 |
| *Sox10* | CAGCCACGAGGTAATGTCCAA | | | | GTGTAGGCGATCTGGGAAGTG | NM_011437 |
| *Sox2* | CCGGACCGCGTCAAGAG | | | | TCATGAGCGTCTTGGTTTTCC | NM_011443 |
| *Sox9* | ACCCACCACTCCCAAAACC | | | | GATGCCGTAACTGCCAGTGTAG | NM_011448 |
| *TrkC* | GGGTCCTGTGGCTGTTATCAG | | | | GGCTCCCTCACCCAATTCTC | NM_008746 |
| *Twist* | GACGAGCTGGACTCCAAGATG | | | | GCCCCTCTGGGAATCTCTGT | NM_011658 |
| *Vimentin* | CCAGAGAGAGGAAGCCGAAA | | | | TTCATACTGCTGGCGCACAT | NM_011701 |
| **Q-RT-PCR primers** | | | |  |  |  |
| **Gene** | | **Forward primer**  **(5’🡪3’)** | | | **Reverse primer**  **(5’🡪3’)** | **GenBank  Accession**  **number** |
| *Calponin* [1] | | ATGCCCAGACCTGGCTCAAA | | | ACTGCAGATGGGCACCAACA | NM_009922 |
| *GAPDH* | | GGGAAGCCCATCACCATCT | | | GCCTCACCCCATTTGATGTT | NM_008084 |
| *Klf4* | | AGACATCGCCGGTTTATATTGAA | | | AACCAAAACCCCCAGATTGC | NM_010637 |
| *Myocardin* [1] | | ACACTCCTGGGGTCTGAACA | | | GCGGTATTAAGCCTTGGTTAGC | NM_145136 |
| *Nanog* | | AAGCGGTGGCAGAAAAACC | | | GTGCTGAGCCCTTCTGAATCA | NM_028016 |
| *Sm22-alpha* [1] | | CAACAAGGGTCCATCCTACGG | | | ATCTGGGCGGCCTACATCA | NM_178598 |
| *Sma* [1] | | TACATGGCGGGGACATTGAA | | | CCGATAGAACACGGCATCATCA | NM_007392 |
| *SMHC* [1] | | AAGCTGCGGCTAGAGGTCA | | | CCCTCCCTTTGATGGCTGAG | NM_013607 |
| *SRF* | | GGCCCCACAGCAAGCGTCTC | | | GTGGCGGGCAACGTCACTGT | NM_020493 |
