# Supplementary material for: Isolation and Characterization of Neural Crest-Derived Stem Cells from Dental Pulp of Neonatal Mice
Source: PLoS One. 2011 Nov 8;6(11):e27526. doi: 10.1371/journal.pone.0027526 (PMC3210810; doi:10.1371/journal.pone.0027526)
Supplement: Table S3 — Lists of differentiation media. (DOC) [file pone.0027526.s013.doc]

**Table S3. Lists of differentiation media**

| **Differentiation** | **Cell density**  **(cells/cm^2^)** | **Media composition** | **Reference** |
| --- | --- | --- | --- |
| Osteogenic  (Osteo-odontogenic) | 2x10^4^cells plated on plastic surface | Serum-free media supplemented with 10% FBS, 10mM β-glycerophosphate (CalBiochem), 0.2mM L-ascorbic acid, and 100nM dexamethasone | [2] |
| Chondrogenic | 2x10^4^cells plated on plastic surface | Serum-free media supplemented with 10% FBS, 100ng/ml BMP-2 (Shenandoah Biotech), 0.2mM L-ascorbic acid, and 100nM dexamethasone | [3] |
| Adipogenic | 2x10^4^cells plated on plastic surface | Serum-free media supplemented with 10% horse serum, 100µM indomethacin (Alfa Aesar), 0.5mM 3-isobutyl-1-methyl-xanthine (ACROS), and 1µM dexamethasone | [4] |
| Neurogenic | 1x10^4^ cells plated on glass surface coated with 5ng/ml fibronectin | Serum-free media with three series of growth factors; 1) media with 100ng/ml bFGF for the first week; 2) media with 10ng/ml FGF-8b and 100ng/ml Shh for the second week; and 3) media with 10ng/ml BDNF (all from R&D) and 10ng/ml EGF (Sigma) for the last week of culture | [5] |
| Smooth muscle | 1.5x10^4^ cells plated on plastic surface coated with 5ng/ml fibronectin | Serum-free media supplemented with 100ng/ml PDGF-BB (R&D) | [6] |
