# Supplementary material for: Isolation and Characterization of Neural Crest-Derived Stem Cells from Dental Pulp of Neonatal Mice
Source: PLoS One. 2011 Nov 8;6(11):e27526. doi: 10.1371/journal.pone.0027526 (PMC3210810; doi:10.1371/journal.pone.0027526)
Supplement: Table S4 — Lists of antibody for staining. (DOC) [file pone.0027526.s014.doc]

**Table S4**. Lists of antibody for staining

| **Marker** | **Antibody** | **Species** | **Dilution** | **Company** |
| --- | --- | --- | --- | --- |
| β-galactosidase | Polyclonal | Rabbit | 1:200 | Invitrogen / Abcam |
| BSP (cells) | Monoclonal | Mouse | 1:100 | Developmental Hybridoma Bank |
| BSP (tissues) | Polyclonal | Rabbit | 1:200 | Renny Franceschi, School of Dentistry, University of Michigan |
| Calponin | Monoclonal | Mouse | 1:100 | Sigma |
| Caldesmon | Monoclonal | Mouse | 1:100 | Sigma |
| CD105-PE | Monoclonal | Rat | 1:50-1:100 | eBioscience |
| CD31-FITC | Monoclonal | Rat | 1:100 | eBioscience |
| CD44-PE | Monoclonal | Rat | 1:50-1:100 | eBioscience |
| CD73-PE | Monoclonal | Rat | 1:50-1:100 | eBioscience |
| COLII | Monoclonal | Mouse | Whole supernatant | Developmental Hybridoma Bank |
| DMP1 | Polyclonal | Rabbit | 1:400 | Takara |
| DSP | Polyclonal | Rabbit | 1:200 | Larry Fisher, NIDCR/NIH |
| GABA | Monoclonal | Rabbit | 1:500 | Sigma |
| KLF4 | Polyclonal | Rabbit | 1:250 | Abcam |
| MSI1 | Polyclonal | Rabbit | 1:250-1:500 | Abcam |
| N-CAM | Monoclonal | Rat | 1:100 | BD PharMingen |
| NF-160/200 | Monoclonal | Mouse | 1:500 | Abcam |
| NG2 | Polyclonal | Rabbit | 1:400 | Abcam |
| OCN | Polyclonal | Rabbit | 1:1000 | Takara |
| OPN | Monoclonal | Mouse | 1:400 | Developmental Hybridoma Bank |
| S100 | Polyclonal | Rabbit | 1:400 | Dako |
| SMA-Cy3 | Monoclonal | Mouse | 1:400 | Sigma |
| SMA-FITC | Monoclonal | Mouse | 1:400 | Sigma |
| Smooth muscle  myosin heavy chain | Monoclonal | Mouse | 1:100 | Sigma |
| SOX10 | Polyclonal | Rabbit | 1:400 | Abcam |
| VEGFR-3 | Monoclonal | Rat | 1:100 | eBioscience |
