# Supplementary material for: Isolation and Characterization of Neural Crest-Derived Stem Cells from Dental Pulp of Neonatal Mice
Source: PLoS One. 2011 Nov 8;6(11):e27526. doi: 10.1371/journal.pone.0027526 (PMC3210810; doi:10.1371/journal.pone.0027526)
Supplement: Text S1 — Supporting Information Methods and Supporting Information Reference. (DOC) [file pone.0027526.s015.doc]

**Text S1. Supporting Information Methods and Supporting Information References**

**Supporting Information Methods**

**RT-PCR and Q-RT-PCR analyses**

cDNA (20ng) was diluted in a final volume of 20µl per reaction using the Immomix PCR Mastermix from Bioline. PCR was performed using the following thermal cycling conditions; 95°C 7 min for initial activation followed by 95°C/30s; 57°C/30s; 72°C/45s, for 35 cycles, with a final 5-min extension at 72°C. Mouse-specific primers are listed in Table S2. Glyceraldehyde-3-phosphate dehydrogenase (*GAPDH*) was utilized as control housekeeping gene. RNA extracted from mouse embryonic stem cells (mESCs), salivary gland, brain, mandible, femur, adipose tissue, calvarial bone tissues and cells (a gift from Dr. Thanaphum Osathanon, Chulalongkorn University, Thailand), and cementoblast cell line (a gift from Dr. Martha Somerman, University of Washington) were used as positive controls while negative controls lacked cDNA.

cDNA (20ng) for Q-RT-PCR were prepared using the SYBR green PCR master mix from Applied Biosystems. Reactions were processed by the ABI 7900HT PCR system with the following parameters: 50°C/2 min and 95°C/10 min, followed by 40 cycles of 95°C/15 s and 60°C/1 min. Results were analyzed using SDS 2.2 software and relative expression calculated using the comparative Ct method. Each sample was run in triplicate reactions for each gene. Error bars represent the standard deviation calculated from the triplicate analysis of each sample or differentiation. cDNA of mESCs and mouse smooth muscle cells (a gift from Dr. William Mahoney Jr., University of Washington) were used to calibrate samples.

***In vitro*** **multi-differentiation**

The compositions of each differentiation media and cell densities are listed in Table S3. Each differentiation media comprised of serum-free media supplemented with specific growth factors for each respective differentiation lineage. The serum-free media was made using the same components and concentrations as stem cell media but without fetal calf serum, PDGF-BB, EGF, and LIF.

**Supporting Information References**

1. Grabski, AD, Shimizu T, Deou J, Mahoney WM, Jr., Reidy MA, et al. (2009) Sphingosine-1-phosphate receptor-2 regulates expression of smooth muscle alpha-actin after arterial injury. Arterioscler Thromb Vasc Biol 29: 1644-1650.

2. Gronthos, S, Graves SE, Ohta S,Simmons PJ (1994) The STRO-1+ fraction of adult human bone marrow contains the osteogenic precursors. Blood 84: 4164-4173.

3. Tscheudschilsuren, G, Bosserhoff AK, Schlegel J, Vollmer D, Anton A, et al. (2006) Regulation of mesenchymal stem cell and chondrocyte differentiation by MIA. Exp Cell Res 312: 63-72.

4. Gregoire, FM (2001) Adipocyte differentiation: from fibroblast to endocrine cell. Exp Biol Med (Maywood) 226: 997-1002.

5. Jiang, Y, Henderson D, Blackstad M, Chen A, Miller RF, et al. (2003) Neuroectodermal differentiation from mouse multipotent adult progenitor cells. Proc Natl Acad Sci U S A 100 Suppl 1: 11854-11860.

6. Ross, JJ, Hong Z, Willenbring B, Zeng L, Isenberg B, et al. (2006) Cytokine-induced differentiation of multipotent adult progenitor cells into functional smooth muscle cells. J Clin Invest 116: 3139-3149.

7. Chen, Z, Couble ML, Mouterfi N, Magloire H, Chen Z, et al. (2009) Spatial and temporal expression of KLF4 and KLF5 during murine tooth development. Arch Oral Biol 54: 403-411.

8. Cheung, M, Chaboissier MC, Mynett A, Hirst E, Schedl A, et al. (2005) The transcriptional control of trunk neural crest induction, survival, and delamination. Dev Cell 8: 179-192.

9. Fong, H, Foster BL, Sarikaya M,Somerman MJ (2009) Structure and mechanical properties of Ank/Ank mutant mouse dental tissues--an animal model for studying periodontal regeneration. Arch Oral Biol 54: 570-576.
